# Immunoglobulin A carries sulfated and *O*-acetylated *N*-glycans primarily at the tailpiece site – strategies for site-specific *N*-glycan identification

**DOI:** 10.3389/fmolb.2025.1595173

**Published:** 2025-08-01

**Authors:** Frania J. Zuniga-Banuelos, Greta Lemke, Marcus Hoffmann, Udo Reichl, Erdmann Rapp

**Affiliations:** ^1^ Bioprocess Engineering, Max Planck Institute for Dynamics of Complex Technical Systems, Magdeburg, Germany; ^2^ glyXera GmbH, Magdeburg, Germany; ^3^ Bioprocess Engineering, Otto von Guericke University Magdeburg, Magdeburg, Germany

**Keywords:** immunoglobulin A (IgA), mass spectrometry, glycoproteomics, *N*-glycosylation, oxonium marker ions, sulfated *N*-glycans, *O*-acetylated *N*-glycans, rare *N*-glycans

## Abstract

Sulfated *N-*glycans from human immunoglobulin A (IgA) were recently discovered via glycomic approaches. However, their site-specific description is still pending. Certain *N-*glycan structures at specific *N-*glycosylation sites in IgA are crucial for microbial neutralization and effector functions. For instance, sialylated *N-*glycans on the C-terminal tailpiece mediate anti-viral activity by interfering with sialic-acid-binding viruses. Sulfated *N-*glycan epitopes can be ligands for viral proteins and thus play a role in the immune response. In this study, we performed a site-specific screening for sulfated and other rare *N-*glycans in two commercially available human serum IgA samples employing an in-depth *N-*glycoproteomic approach, previously developed by us. We found evidence of complex-type and hybrid-type *N-*glycans containing sulfated *N-*acetylhexosamine (sulfated HexNAc) attached to the *N-*glycosylation sites in the tailpiece and the C_H_2 domain of both IgA subclasses. Also, complex-type *N-*glycan compositions bearing *O-*acetylated sialic acid were identified primarily at the tailpiece site. Surprisingly, *N-*glycans bearing glucuronic acid were identified in the commercial IgA samples, but from peptides of “contaminant” glycoproteins. A detailed comparison of the *N-*glycosylation profiles of human serum IgA samples from two suppliers showed such *N-*glycans with sulfated HexNAc consistently in higher abundance in the tailpiece region. These findings have not been described before for a site-specific glycopeptide analysis. Overall, our work provides strategies for performing a dedicated site-specific search for sulfated and *O-*acetylated *N-*glycans that can be easily transferred, e.g., to human IgA derived from mucosal tissues, milk, or saliva. We expect that a wider and deeper micro-heterogeneity description of clinically relevant glycoproteins, such as immunoglobulins, can expand the screening for biomarkers or treatment options.

## Graphical Abstract

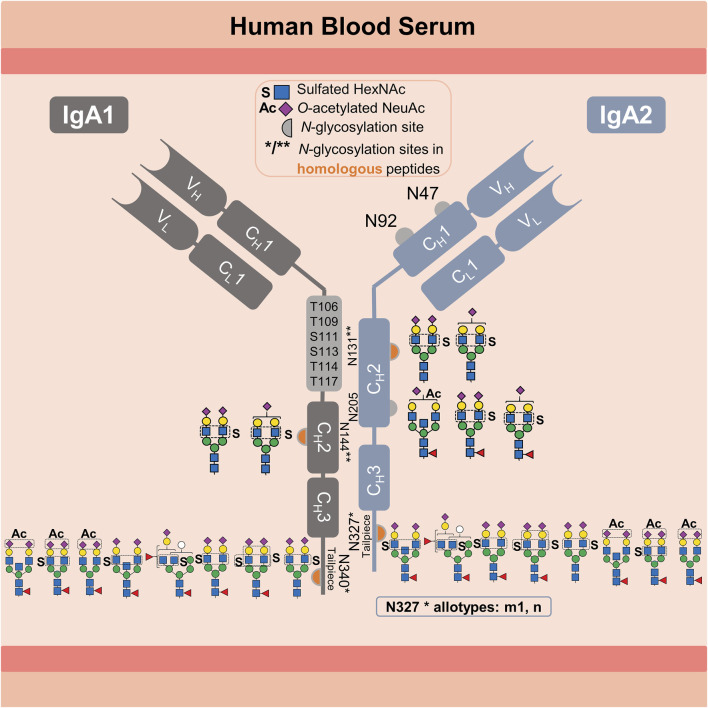

## 1 Introduction

Human immunoglobulin A (IgA) is the most abundant secretory immunoglobulin and the second most abundant in serum (Mestecky et al., 1989; Kerr, 1990). IgA plays a crucial role in the defense against mucosal infections, microbiota modulation, and newborn immunization (Ruiz-Palacios et al., 1990; Jorgensen et al., 2013; Takeuchi and Ohno, 2022). Due to its potent antiviral activity, tumor cell-killing effector mechanism and ability to inhibit inflammatory and autoimmune diseases, IgA has an enormous therapeutic potential (Huls et al., 1999; Monteiro, 2010; Maurer et al., 2018). In humans, IgA exists in two subclasses, IgA1 and IgA2 (ratio 89 to 11 in serum) (Kerr, 1990). Variants with amino acid substitutions exist for both subclasses (e.g., IgA2 M319L related to UniProt P01877 and P0DOX2). IgA2 exists in three genetically controlled polymorphisms (allotypes) m1, m2, and n, whose ratio in serum varies among different ethnical groups (Torano and Putnam, 1978; Tsuzukida et al., 1979; Chintalacharuvu et al., 1994). The highly *O-*glycosylated hinge region of IgA1 is 13 amino acids longer than the IgA2 hinge region (UniProt P01876) (Kerr, 1990). IgA1 and IgA2 are present in two structural conformations: as dimer or monomer. In dimeric conformation, transport across the epithelial barriers is mediated by binding to the polymeric immunoglobulin receptor (pIgR), the precursor of the secretory component required to form secretory IgA (Kerr, 1990). Between 87% and 97% of IgA secreted into mucosal tissues (intestinal, nasal, and oral tissues) are dimerized via the joining-chain (J-chain). In contrast, between 85% and 99% of the IgA present in blood serum exist as monomers (Mestecky et al., 1989). Both IgA subclasses are modified with *N-*glycans and share homologous sequences around the *N-*glycosylation sites in the C_H_2 domain (N144-IgA1/N131-IgA2), as well as on the C-terminal tailpiece in the C_H_3 domain (N340-IgA1/N327-IgA2_m1/n_). However, the m2 allotype is not homologous to the m1 and n allotypes in the tailpiece sequence (Kerr, 1990; Chintalacharuvu et al., 1994). All IgA2 allotypes have two additional *N-*glycosylation sites, one in the C_H_2 domain and another in the C_H_1 domain (N205 and N47), but only the allotypes m2 and n have a fifth *N-*glycosylation site (N92) in the C_H_1 domain (Kerr, 1990; Chintalacharuvu et al., 1994). For better clarity, the graphical abstract provides an overview of the information presented above.

The *N-*glycosylation profile varies between IgA subclasses and *N-*glycosylation sites. A lectin blot analysis of each serum IgA subclass revealed that IgA2 has fewer sialylated, galactosylated and bisected *N-*glycans, but more hybrid- and oligomannose-type *N-*glycans compared to IgA1 (Steffen et al., 2020). These specific *N-*glycosylation profiles are critical for the pro-inflammatory effect of IgA2 and the anti-inflammatory modulatory role of IgA1 (Steffen et al., 2020). Additionally, several studies have used bottom-up mass spectrometry analyses to obtain a site-specific description of the serum IgA *N-*glycosylation (Gomes et al., 2008; Bondt et al., 2016; Plomp et al., 2018; Chandler et al., 2019; Steffen et al., 2020; Clerc et al., 2023). However, the *N-*glycans observed at the N144-IgA1 and N131-IgA2 sites (in the C_H_2 domain), as well as the tailpiece sites N340-IgA1 and N327-IgA2_m1/n_ (in the C_H_3 domain), are reported for both IgA subclasses due to the homologous sequences surrounding these sites. Yet, significant differences between the *N-*glycosylation profile of both C_H_2 and C_H_3 domains were observed. The C_H_2 domain features mostly non-fucosylated hybrid-type *N-*glycans and di-antennary complex-type *N-*glycans with terminal galactose or sialic acid. In contrast, the tailpiece typically bears fucosylated di- or multi-antennary complex-type *N-*glycans, which are variably sialylated and bisected, as well as oligomannose-type *N-*glycans. Some studies show that *N-*glycans attached to the sites in the C_H_2 domain and the tailpiece (IgA1 Fc *N-*glycans) are irrelevant for binding to the FcαRI receptor (Mattu et al., 1998; Gomes et al., 2008). However, other studies indicate that IgA1 Fc *N-*glycans are relevant for other receptors involved in anti-inflammatory mechanisms (Monteiro, 2010; Steffen et al., 2020). Closer investigations on the *N-*glycosylation at the tailpiece have demonstrated its high impact on different IgA functions. First, *N-*glycosylation at the tailpiece is critical for binding to complement C3 protein (Chuang and Morrison, 1997). Second, it was shown to be important for modulating dimer formation in IgA1 (Chuang and Morrison, 1997). Third, *N-*glycosylation at the tailpiece enhances the IgA anti-viral neutralizing activity by exposing sialylated *N-*glycans and interacting with sialic-acid-binding viral proteins, like influenza hemagglutinin and neuraminidase (Maurer et al., 2018). Fourth, it affects the serum half-life of IgA1 (Rifai et al., 2000). Rifai et al. showed that the clearance of IgA1 from serum is slower when *N-*glycans at the tailpiece are removed, whereas it remained unchanged when *O-*glycans are removed (Rifai et al., 2000). Although the function of IgA2 *N-*glycans on the C_H_1 domain (Fab region) remains unclear so far, Rifai et al. suggest that the additional *N-*glycosylation sites on IgA2 (N47 and N92 in the C_H_1 and N205 in C_H_2) alter the rate of IgA2 clearance from blood. These findings demonstrate the significance of elucidating protein *N-*glycosylation in terms of micro-heterogeneity (describing the site-specific *N-*glycan variability), for the comprehension of its role in the mechanisms modulating the effector functions of IgA.

The complexity of IgA *N*-glycosylation is further increased by a significant number of *N-*glycans that are modified by sulfation. This was detected for the first time in 1999 by Boisgard et al. in IgA from mammary glands of rabbit in addition to other species and tissues (Boisgard et al., 1999). However, it was only confirmed for human serum IgA 20 years later through in-depth glycomic analyses (Chuzel et al., 2021; Cajic et al., 2023; Burock et al., 2025). Identification of sulfated *N-*glycans is a challenging task. In several other glycomic and glycoproteomic analyses conducted on IgA, sulfated *N-*glycans never appeared in the identification lists (Gomes et al., 2008; Bondt et al., 2016; Plomp et al., 2018; Chandler et al., 2019; Goonatilleke et al., 2019; Steffen et al., 2020; Clerc et al., 2023). Recently Cajic et al., Chuzel et al., and Burock et al., confirmed the presence of the sulfated *N-*glycan FA2G2S2-SO_4_ in human serum IgA (Chuzel et al., 2021; Cajic et al., 2023; Burock et al., 2025). Chuzel et al. demonstrated that the sulfate was linked to the 6-carbon of GlcNAc (GlcNAc-6-SO_4_) by using a highly-specific sulfatase in combination with the methodology developed by Cajic et al. (Chuzel et al., 2021). In the study from Cajic et al., the sulfated *N-*glycan FA2G2S2-SO_4_ was released upon cleavage of IgA *N-*glycans and all together were labeled with the removable fluorescent dye 9-fluorenylmethyl chloroformate (Fmoc) (Cajic et al., 2023). The Fmoc-labeled sulfated *N-*glycan was isolated by hydrophilic interaction high-performance liquid chromatography (HILIC-HPLC), identified, and characterized employing two orthogonal approaches: one based on matrix-assisted laser desorption/ionization time-of-flight mass spectrometry (MALDI-TOF-MS) and another based on multiplexed capillary gel electrophoresis with laser-induced fluorescence detection (xCGE-LIF) *N-*glycan analysis (Cajic et al., 2023). Cajic et al. demonstrated that the newly discovered sulfated *N-*glycan, with the composition HexNAc_4_Hex_5_Fuc_1_NeuAc_2_Sulfo_1_, presents α2-6-Neu5Ac on both antennae and GlcNAc-6-SO_4_ on the α1-3-Man arm. The highly-specific sulfatase has been applied to elucidate sulfated *N*-glycans released from other proteins (Burock et al., 2025). Overall, Cajic et al., Chuzel et al., and Burock et al. definitely demonstrated that human IgA samples obtained from diverse manufacturers and batches reproduce the same finding (Chuzel et al., 2021; Cajic et al., 2023; Burock et al., 2025). Though the specific glycosylation sites could not be determined (Cajic et al., 2023). Alagesan et al. reported other sulfated *N-*glycans bearing sulfated galactose, which were associated with the heavy chains of both human IgA subclasses but not assigned to specific *N-*glycosylation sites (Alagesan et al., 2019). These galactose sulfated *N-*glycans were deposited in GlyConnect (GlyConnect, 2019a; GlyConnect, 2019b). Although the role of sulfated *N-*glycans in IgA still remains largely unexplored, it is evident that the role of sulfated glycans in general is not minor, since they impact cell-cell interaction and, as HexNAc-sulfated sialosides, can be ligand for some influenza hemagglutinin variants (Lo-Guidice et al., 1994; Mowery et al., 2004; Wang et al., 2009; Liao et al., 2010; Otsuki et al., 2010). Thus, despite their relevance, the site-specific detection of the low-abundant sulfated *N-*glycans by mass spectrometry is still pending. This is due to their even lower abundance per glycosylation site, inadequate data analysis, and limitations associated with mass spectrometry (MS) measurement. The negative charge on sulfated sugars causes low ionization efficiency and instability of the sulfated fragment ions (Shi et al., 2012).

In the present study we determined the *N-*glycosylation sites of human IgA that harbor sulfated *N-*glycans using an in-depth *N-*glycoproteomic approach. For the first time, this allowed the site-specific identification of HexNAc-sulfated *N-*glycans in human IgA. The approaches developed by us in previous works allow the identification of intact *N-*glycopeptides from the low-abundant *N-*glycoproteome including *N-*glycopeptides that feature sulfated, glucuronidated, or *O-*acetylated *N-*glycans (Hoffmann et al., 2018; Zuniga-Banuelos et al., 2025). The present study reveals that sulfated *N-*glycans are linked to the C_H_2 domain sites N205, N131 in IgA2 and N144 in IgA1, as well as in the tailpiece site N340 in IgA1 and N327 in IgA2_m1/n_. It was observed that the IgA C-terminal tailpiece shows the highest abundance of sulfated *N-*glycans. The abundance of sulfated *N-*glycans substantially differs comparing two commercially available human serum IgA samples. In addition to the sulfated *N-*glycan FA2G2S2-SO_4_ (HexNAc_4_Hex_5_Fuc_1_NeuAc_2_Sulfo_1_) described by our previous *N-*glycomic analyses (Chuzel et al., 2021; Cajic et al., 2023), here also other *N-*glycan compositions carrying sulfated HexNAc and *O-*acetylated sialic acid were identified on human IgA. *O-*acetylated *N-*glycans have been also previously characterized in our group by Cajic et al. (Cajic et al., 2023), but in horse serum. In the future, the methodology we have developed here will allow site-specific examination of sulfated and *O*-acetylated *N-*glycans in IgA extracted from other body fluids (e.g., saliva or milk) (Goonatilleke et al., 2019). Longitudinal studies that integrate IgA rare *N-*glycans can be beneficial in various clinical conditions such as inflammatory bowel diseases or rheumatoid arthritis (Clerc et al., 2023; Mayboroda et al., 2023; Xu et al., 2023). Expanding the research on the role of sulfated and *O*-acetylated *N-*glycans in IgA effector function can determine whether these rare *N-*glycans are relevant as a critical quality attribute (CQA) of recombinantly expressed IgA for therapeutic use. Finally, this work also applies strategies to maximize the identification of rare *N-*glycan compositions, necessary to identify the *N-*glycopeptides of interest in IgA samples. These strategies can be adapted to other instances, and they are particularly useful in cases where the identification of *N-*glycopeptides is hindered by a lack of knowledge about the peptide and *N-*glycan components.

## 2 Materials and methods

### 2.1 Samples and materials

Commercial human IgA purified from blood serum was purchased from two suppliers: supplier 1, Sigma-Aldrich (14036-1 MG, St. Louis, MO, United States); and supplier 2, Athens Research and Technology (16-16-090701, Athens, Georgia, United States). Milli-Q water suitable for LC-MS analysis was freshly obtained from Millipore Milli-Q® Advantage A10 system (18,2 MΩ × cm, <5 ppb) equipped with a LC-Pak® Polisher filter unit (#LCPAK0001) purchased from Merck Millipore (Darmstadt, Germany). The reagents applied were MS grade or the highest purity available. LC-MS grade acetonitrile (ACN, A955-212) and trifluoroacetic acid (TFA, 28904) were purchased from Fisher Scientific (Schwerte, Germany). Ammonium bicarbonate (ABC, 09830), formic acid (FA, 56302), DL-dithiothreitol (DTT, D5545), iodoacetamide (IAA, I1149) and calcium chloride (CaCl_2_, A4689) were purchased from Merck (Darmstadt, Germany). Sequencing grade trypsin LC-MS grade was purchased from Promega (#V5111, Madison, WI, United States).

### 2.2 Digestion

**FIGURE 1:**
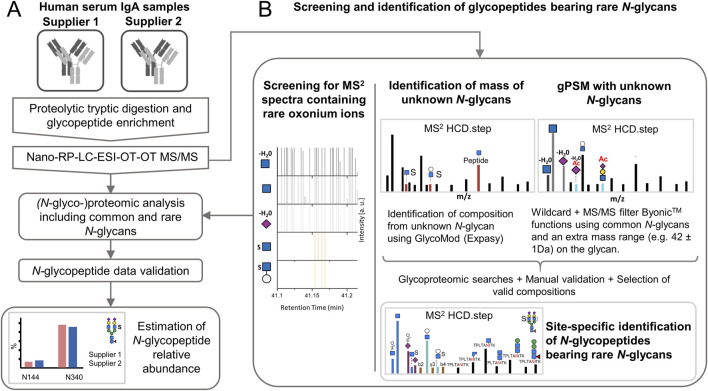
Experimental design for expanding the *N*-glycoproteomic analysis of IgA by applying strategies for the identification of sulfated and other rare *N-*glycopeptides. **(A)** Analysis of human serum IgA of two commercial suppliers where the identification of rare *N-*glycopeptides is integrated. **(B)** Strategies to determine the software search parameters for maximizing the identification of rare *N-*glycopeptides. Nano-RP-LC-ESI-OT-OT-M/MS: nano-reversed phase-liquid chromatography with electrospray-ionization coupled orbitrap tandem mass spectrometric measurement for precursor and fragment ions. HCD.step: higher-energy collisional dissociation with stepped normalized collisional energy [NCE of 20, 35, and 50]. gPSM: glycopeptide spectra match.

The sample preparation workflow is depicted in Figure 1A and operational features are summarized in Table 1. The proteolytic digestion of two commercial human serum IgA samples (supplier 1 and supplier 2) was conducted as described by Hoffmann et al. (2018). Using the filter aided sample preparation approach (FASP) developed by Wisniewski et al. (2009) and modified by Hoffmann et al. (2018). A protein amount of 100 μg per IgA sample was loaded on the 10 kDa Nanosep® Omega Filters (OD010C35, Pall®). For digestion, the ratio set was 1 μg of trypsin per 60 μg of protein sample. After adding trypsin, the sample fractions were incubated over night at 37°C and 300 rpm. To recover the eluates, the filters were centrifuged at 10,000 x g for 15 min (set up applied for all the following steps). The filter membrane was washed with 50 μL of 50 mM ABC_(aq)_ with 5% (v/v) ACN and then with 50 μL of water. The eluates were dried in a rotational vacuum concentrator (0.01 mbar, ca. 3 h, 1°C, same parameters were applied for all drying steps).

**TABLE 1:** Summary of operational features and parameters used for sample preparation and LC-MS/MS acquisition.

Workflow step	Parameter	Description
Proteolytic digestion	Reference	Wisniewski et al. (2009), Hoffmann et al. (2018)
	Method	FASP
	Proteolytic enzyme	Sequencing grade trypsin
	Digestion conditions	Ratio 1 µg enzyme per 60 µg protein Buffer 50 mM NH_4_HCO_3(aq)_ + 1 mM CaCl_2(aq)_ + 5% (v/v) ACN_(aq)_ 37°C overnight 300 rpm
Glycopeptide enrichment	Reference	Selman et al. (2011), Zuniga-Banuelos et al. (2025)
	Enrichment method	Cotton-hydrophilic interaction liquid chromatography-solid phase extraction microtips
	Fractionation steps	Depletion, wash, elution 1 and elution 2 (collected in separated 0.5 mL tubes)
	Equilibration of microtips	Wash pipetting 3 times 100 µL water and equilibrate pipetting 5 times 100 µL 85% ACN + 0.1% TFA_(aq)_
	Loading mobile phase	Resuspend lyophilized peptides in 50 µL 85% (v/v) ACN_(aq)_ and pipette up and down 20 times
	Wash mobile phase	Pipette 3 times 100 µL 85% (v/v) ACN_(aq)_ + 0.1% (v/v) TFA_(aq)_
	Elution 1 mobile phase	Pipette 3 times 100 µL 78% (v/v) ACN_(aq)_ + 0.1% (v/v) TFA_(aq)_
	Elution 2 mobile phase	Pipette 4 times 100 µL H_2_O
MS acquisition	Reference	Hoffmann et al. (2018)
	Ionization mode	Positive ion mode
	Voltage	2550 V
	Ion transfer temperature	275°C
	Data dependent acquisition	Cycle time:1.5 s
	Sweep Gas (Arb)	0
	Auxiliar Gas (Arb)	0
	Sheat Gas (Arb)	0
	Precursor ion scan	Orbitrap resolution: 120,000
		Quadrupole isolation: true
		RF Lens: 30%
		Scan range: 350–1,500 m/z
		AGC target: standard
		Maximum injection time: 50 ms
	Filters	Monoisotopic peak determination: peptide
		Include charge states: 2-7
	Dynamic Exclusion	Exclude after n times: 1
		Exclusion duration: 15 s
		Mass tolerance: ±10 ppm
		Exclude isotopes: true
	Fragment ion scan	Orbitrap resolution: 60,000
		Isolation mode: quadrupole
		Isolation window: 1.6 m/z
		Isolation offset: off
		Activation type: HCD
		Collision energy mode: stepped
		HCD collision energy type: normalized
		HCD collision energies %: 20, 35, 50
		Scan range mode: auto
		AGC target: standard
		Maximum injection mode: custom
		Maximum injection time: 118 ms
		Data type: Centroid
	Targeted mass trigger	m/z list: 284.0435, 446.0963, 336.1395 (z = 1)
		Trigger only with detection of at least 2 ions from the list
		Mass tolerance ±10 ppm
	Triggered fragment ion scan	Orbitrap resolution: 60,000
		Isolation mode: quadrupole
		Isolation window: 1.6 m/z
		Isolation offset: off
		Activation type: HCD
		Collision energy mode: fixed
		HCD collision energy type: normalized
		HCD collision energies %: 20
		Scan range mode: auto
		AGC target: standard
		Maximum injection mode: custom
		Maximum injection time: 118 ms
		Data type: Centroid

### 2.3 Cotton-hydrophilic interaction liquid chromatography-solid phase extraction (cotton-HILIC-SPE)

The glycopeptide enrichment method applied here (Zuniga-Banuelos et al., 2025) is a modification of the method from Selman et al. (2011). The tryptic peptides from the IgA samples (from supplier 1 and 2) were resuspended in 100 μL 85% (v/v) ACN_(aq)_. For each sample, the glycopeptide enrichment was conducted by four replicates using 20 μL of tryptic peptides each time. Four HILIC-fractions were collected: depletion, wash, elution 1, and elution 2. Depletion and wash fractions were pooled since both contain almost no glycopeptides. All HILIC-fractions were dried, stored at −20°C, and resuspended in 10 μL water on the day of injection.

### 2.4 LC-MS/MS analysis

Nano-reversed-phase liquid chromatography coupled to electrospray ionization orbitrap tandem mass spectrometry (nanoRP-LC-ESI-OT-OT-MS/MS) was conducted using a Dionex UltiMate 3000 RSLCnano system (UHPLC, Thermo Fisher Scientific, Germering, Germany) coupled to an Orbitrap Eclipse Tribrid mass spectrometer. A C18 trap column (length 2 cm, pore size 100 Å, particle size 5 μm, inner diameter 100 μm, Acclaim PepMap™ 100, #164199 Thermo Fisher Scientific, Lithuania), together with a C18 separation column (length 25 cm, pore size 100 Å, inner diameter 75 μm, particle size 2 μm, Acclaim PepMap™ RSLC nanoViper #164941, Thermo Fisher Scientific, Lithuania) were used for sample separation in the UHPLC system. For the run, 3 μL of sample were injected using the loading pump (100% mobile phase A: 0.1% (v/v) FA_(aq)_) and a flow rate of 7 μL/min. Five minutes after injection the trap column was switched in line with the separation column. The nano pump was operated at a flow rate of 300 nL/min at 40 °C using mobile phase A (0.1% (v/v) FA_(aq)_) and mobile phase B (80% (v/v) ACN_(aq)_ + 0.1% (v/v) FA_(aq)_) to obtain the following separation gradient: 5% B (0–5 min); 5%–31% (5–35 min); 31%–44% (35–40 min); 44%–95% (40–41 min); 95% (41–46 min); 95%–5% (46–47 min); 5% (47–60 min). The ion sources spray voltage was static set to 2.55 kV and the temperature of the ion transfer tube was set to 275°C.

Mass spectrometric data acquisition of precursor (MS or MS^1^) and fragment ion spectra (MS/MS or MS^2^) was conducted with an Orbitrap Eclipse Tribrid mass spectrometer (OT-OT-MS/MS; Thermo Fisher Scientific, Dreieich, Germany) at higher-energy collisional dissociation (HCD) with stepped normalized collisional energy (NCE 20, 35, 50%) “HCD.step”, as described by Hoffmann et al. (2018). MS precursor ion scans were performed in positive ion mode using a scan range of m/z 350 to 1,500. The automatic gain control (AGC) target was set to “standard” with a resolution of 120,000 and a maximum injection time of 50 ms. Dynamic exclusion was set as follows: exclusion duration to 15 s, repeat count one, and mass tolerance of 10 ppm. Data-dependent acquisition mode with 1.5 s cycle time between precursor spectrum acquisitions. MS data-dependent fragment ion scans were acquired performing HCD.step at a resolution of 60,000, scan range set to “normal” and isolation window set to m/z 1.6.

Additional acquisition of fragment ion scans was triggered by the detection of at least two out of the following three oxonium marker ions: HexNAc_1_Hex_1_ [M+H]^+^/366.1395, HexNAc_1_Sulfo_1_ [M+H]^+^/284.0435, HexNAc_1_Hex_1_Sulfo_1_ [M+H]^+^/446.0963, with mass accuracy of 10 ppm. Also here, an HCD method with a fixed NCE of 20 “HCD.low” was employed at a resolution of 60,000 with a scan range set to “normal” and isolation window of m/z 1.6. Resulting from different fragmentation regimes, HCD.low fragment ion scans complemented the information content of the acquired HCD.step spectra (Hoffmann et al., 2018). Table 1 summarizes MS instrument parameters applied in the present work.

### 2.5 Identification of new *N-*glycan compositions

As shown in Figure 1B, two strategies relying on the characteristic oxonium marker ions were iteratively applied for the identification of new *N-*glycan compositions in acquired glycopeptide MS^2^ spectra. The HexNAc-sulfated oxonium marker ions applied here were: HexNAc_1_Sulfo_1_ [M+H]^+^/284.0435 and Hex_1_HexNAc_1_Sulfo_1_ [M+H]^+^/446.0963, while the NeuAc *O-*acetylated oxonium marker ions used here were: NeuAc_1_Ac_1_ [M-H_2_O+H]^+^/316.1027 and HexNAc_1_Hex_1_NeuAc_1_Ac_1_ [M+H]^+^/699.2455). The first strategy is based on estimating the mass of the unknown *N-*glycan moiety and predict the possible composition (based on oxonium ions observed) with the open source GlycoMod tool from expasy.org (Cooper et al., 2001). The second strategy implements both, glycan “wildcard” search and MS/MS filter Byonic™ functions (Version 5.5.2, Protein Metrics, Cupertino CA, United States).

In the oxonium ions-guided strategy, the MS^2^ spectra acquired from the *N-*glycopeptide enriched samples were screened for oxonium marker ions using a scan filter in Thermo Xcalibur Qual Browser software (Version 2.2, Thermo Fisher Scientific, Bremen, Germany) as described by Zuniga-Banuelos et al. (2025). The mass of the unknown *N-*glycan moieties was estimated for each MS^2^ scan by subtracting the observed putative peptide mass from the precursor mass. The putative peptide mass was determined by relying on the conserved fragmentation pattern: (1) [M_peptide_ + H -NH_3_]^+^, (2) [M_peptide_+H]^+^, (3) [M_peptide_+H+^0.2^X HexNAc]^+^ and (4) [M_peptide_+H+HexNAc]^+^ (NH_3_=17.0265 Da, ^0.2^X HexNAc=83.0371 Da; and HexNAc=203.0794 Da) (Hoffmann et al., 2018).

In the strategy that includes a “wildcard” search and MS/MS filter functions, the software selectively conducts a glycoproteomic wildcard search on MS^2^ spectra containing a defined set of oxonium marker ions. The wildcard search adds masses equivalent to a specific *N-*glycan modification (e.g., sulfate or acetyl group mass) to a set of common *N-*glycans and not to the peptide moiety (Roushan et al., 2021).

Another goal of this work was screening for MS^2^ scans containing other rare *N-*glycans present in the IgA samples, using the oxonium marker ions described by Zuniga-Banuelos et al. (2025). As a result, *N-*glycopeptides presenting the oxonium ion HexNAc_1_Hex_1_HexA_1_ [M+H]^+^/542.1716, where HexA is typically glucuronic acid in human *N-*glycans, were also observed. The correct *N-*glycan compositions were identified by means of the strategies presented above.

### 2.6 *(N-*glyco)proteomic data analysis

This study applied a modified version of the *N-*glycopeptide search pipeline applied by Zuniga-Banuelos et al. (2025). Based on observations obtained from our previous work, it was determined to undertake separated focused *N-*glycopeptide searches that will be outlined below. This strategy represents an advantage, as each search includes only MS^2^ spectra containing features valid for each *N-*glycopeptide composition, (i.e., oxonium marker ions), which reduces the risk of incorrect glycopeptide-spectrum matches (gPSM) and time required for manual validation.

The first (*N-*glyco)proteomic data analysis (Figure 1A) was conducted on all technical replicates from the IgA samples (from two suppliers). The second *N*-glycoproteomic data analysis focuses on sulfated *N*-glycopeptide identification using an alternative search setup, and it is described in the following paragraph. Both, the proteomic and the *N-*glycoproteomic searches included the MS/MS raw data from all HILIC-fractions (depletion/wash, elution 1, and elution 2). For each sample, one proteomic and eight glycoproteomic searches were conducted using Byonic™. The proteomic searches were set using the human canonical proteome UniprotKB (June 2022, 20,386 canonical sequences). From the proteomic search a focus protein database per IgA sample was extracted and set as “focused protein database” file for the glycoproteomic searches. The first four glycoproteomic searches used this focus protein database with four variations on the *N-*glycan composition groups searched, which were: 1) *N-*glycan compositions with common *N-*glycans, 2) *N-*glycan compositions with HexNAc sulfation, 3) *N-*glycan compositions with *O-*acetylated sialic acid, and 4) *N-*glycan compositions with glucuronic acid. Four additional glycoproteomic searches per sample using these *N-*glycan composition groups focused on the identification of *N-*glycopeptides from truncated variants of the IgA1-and IgA2-tailpiece. Each dedicated *N-*glycoproteomic search was strictly conditioned by the presence of the oxonium marker ion characteristic of the *N-*glycan composition groups searched by using the MS/MS filter Byonic™ function. The parameters set for the proteomic and *N-*glycoproteomic searches per supplier sample are shown in Supplementary Table S1. The results from the 18 searches were imported and combined in Byologic™, to create one (*N-*glyco)peptide identification list. The *N-*glycopeptide identifications with a “Byonic MS2 search score” above 100 were manually validated (as described in Zuniga-Banuelos et al. (2025)) and considered for further analyses. In this glycoproteomic analysis, gPSM presenting rare *N-*glycan compositions were accepted as “True” only if their corresponding oxonium marker ions were present in the MS^2^ spectra.

To account for the instability of sulfated fragment ions and the effect of conditioning the identification of sulfated *N-*glycopeptides on the detection of sulfated oxonium ions, a second glycoproteomic data analysis was performed on the MS/MS raw data of all HILIC-fractions from both samples. The analysis included only the searches for sulfated *N-*glycopeptides described in Supplementary Table S1, but without setting HexNAc-sulfated oxonium ions (HexNAc_1_Sulfo_1_ [M+H]^+^ and Hex_1_HexNAc_1_Sulfo_1_ [M+H]^+^) in the MS/MS filter Byonic™ function. The resulting *N-*glycopeptide identification lists were integrated in Byologic™, and manually validated as described in Zuniga-Banuelos et al. (2025). In this instance, gPSM lacking HexNAc-sulfated oxonium marker ions but correctly matching common oxonium ions, b and y fragment ions, and the conserved peptide fragmentation pattern were set as “True”, and considered for further comparative analyses.

### 2.7 Relative quantification of (*N-*glyco)proteomic data

The relative abundance of IgA1 and IgA2 subclasses was calculated on all technical replicates of the IgA samples from both suppliers by including the MS/MS raw files obtained from all HILIC-fractions. Label-free quantification was conducted within a single proteomic analysis using Proteome Discoverer (version 2.5.0.400, Thermo Fisher Scientific, Bremen, Germany). The software setup allowed defining the files by supplier and technical replicates before the analysis. The search engines Sequest HT (Proteome Discoverer 2.5.0.400, Thermo Fisher Scientific, Bremen, Germany) and Mascot (version 2.6, Matrix Science, London, United Kingdom) were set using the human protein database SwissProt/UniprotKB (20,315 canonical sequences, v2022-06-14), full specific tryptic digestion, two missed cleavages allowed, precursor and fragment mass tolerance: 10 ppm and 0.02 Da respectively. The following dynamic modifications were set: deamidated (N, Q), oxidation (M), acetyl (protein N*-*Terminus). Carbamidomethyl (C) was set as static modification. Percolator was applied for peptide validation allowing 1% false discovery rate (FDR) on peptide level. Minora feature detector node, included in the processing workflow, enabled detecting and grouping peptide signals for the HILIC-fractions that belong to the same technical replicate. The label-free quantification applied in the consensus workflow integrated the nodes feature mapped and precursor ions quantifier, considering “unique + razor” peptides with all other parameters set as default.

The proteomic data derived from Proteome Discoverer was processed with Microsoft Excel (2016). The relative abundance of IgA subclasses per replicate was calculated by normalizing the integrated peak area of each representative IgA1- or IgA2-peptide (“NFPPSQDASGDLYTTSSQLTLPATQCLAGK” or “NFPPSQDASGDLYTTSSQLTLPATQCPDGK”, respectively) including variants due to missed cleavages and chemical modifications by the total integrated peak area of both peptides. The values of the technical replicates were averaged for each supplier sample.

Label-free quantification was also carried out on the validated (*N-*glyco)peptide list from the (*N-*glyco)proteomic Byonic™ data analysis. The (*N-*glyco)proteomic data combined in Byologic™ was processed with Microsoft Excel (Professional Plus 2021) for relative quantification of IgA *N-*glycan compositions per site (micro-heterogeneity). The (*N-*glyco)peptides were classified by *N-*glycosylation site, peptide sequence homology within the identified IgA sequences (P01876, P01877, and P0DOX2 Uniprot June 2022), and then grouped by *N-*glycan compositions (neglecting differences caused by peptide modifications and missed cleavages). The *N-*glycan compositions were quantified if at least three technical replicates showed values from valid *N-*glycopeptide identifications. The abundance of each *N-*glycan composition was normalized by the total area under the curve of all *N-*glycan compositions identified per corresponding *N-*glycosylation site. The values of the technical replicate were averaged for each supplier sample.

## 3 Results

Site-specific *N-*glycans on the Fc region from each human IgA subclass play a particular role in the effector function. Our recently published glycomic experiments have demonstrated the presence of the *N-*glycan FA2G2S2-SO_4_ bearing sulfated HexNAc in human serum IgA (Chuzel et al., 2021; Cajic et al., 2023; Burock et al., 2025). In addition, other sulfated and *O-*acetylated *N-*glycans have been associated with human serum IgA (Alagesan et al., 2019; GlyConnect, 2019a; GlyConnect, 2019b; Burock et al., 2025). However, the specific position and the relative abundance of sulfated *N-*glycans in human serum IgA subclasses have not been elucidated so far. In the present study, an in-depth *N-*glycoproteomic workflow, previously established by us (Zuniga-Banuelos et al., 2025), was applied to human serum IgA from two commercial suppliers and optimized for expanding the micro-heterogeneity description of IgA *N-*glycosylation. To this end, human serum IgA samples from two commercial suppliers were tryptically digested and enriched for glycopeptides. Technical quadruplicates were obtained for each commercial sample by repeating the cotton-HILIC-SPE protocol four times. All HILIC-fractions from the technical replicates (depletion/wash, elution 1, and elution 2) were analyzed by nanoRP-LC-ESI-OT-OT-MS/MS (Figure 1A). To identify unknown *N-*glycan compositions, strategies to maximize the identification of rare *N-*glycans were applied to the glycopeptide MS^2^ spectra containing oxonium marker ions of sulfated and *O-*acetylated *N-*glycans (Figure 1B). Also, a diagnostic search for other rare *N-*glycans was conducted based on oxonium ions described by Zuniga-Banuelos et al. (2025). Once the compositions of the unknown *N-*glycan were predicted using the strategies described in Materials and Methods, an expanded *N-*glycoproteomic analysis was conducted. All measurements of the HILIC-fractions were included in the proteomic and *N-*glycoproteomic analyses.

### 3.1 Site-specific identification of HexNAc-sulfated *N-*glycans

To maximize the identification of sulfated *N-*glycopeptides three strategies (screening for MS^2^ spectra containing rare oxonium ions, identification of masses of unknown *N*-glycans, and gPSM with unknown *N*-glycans) were applied to the IgA samples as described in Materials and Methods (also depicted in Figure 1B). This was accomplished primarily through the screening of MS^2^ scans for HexNAc-sulfated oxonium marker ions and conducting multiple iterations of glycoproteomic searches using the different (*N-*glyco)peptide modifications parameters (i.e., *N-*glycan compositions and peptide modifications). Human IgA *N-*glycans reported in Glyconnect database were also considered as a source of sulfated *N-*glycan compositions (Supplementary Table S2) (Alagesan et al., 2019; GlyConnect, 2019a; GlyConnect, 2019b). Once valid gPSMs showing HexNAc-sulfated oxonium marker ions evidence were identified and validated, the parameters of the glycoproteomic search were recorded and integrated to an expanded glycoproteomic search (Supplementary Table S1), which will be further described in the following sections.

**FIGURE 2:**
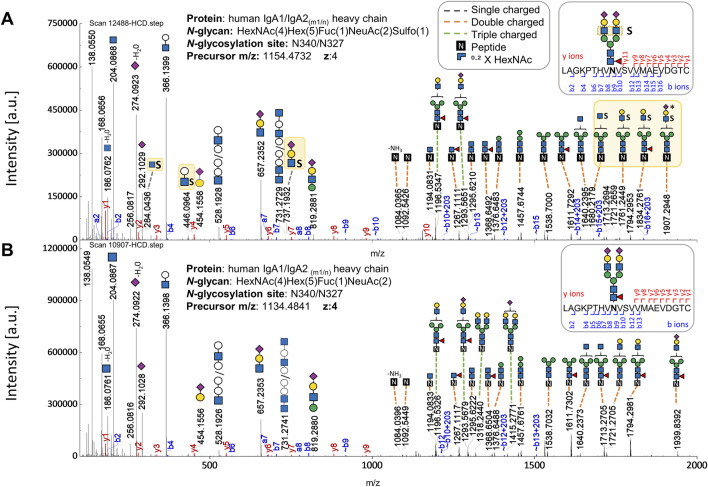
Comparison of HCD.step fragment ion spectra derived from two *N-*glycopeptides (with the same peptide backbone) - one bearing the sulfated versus the one bearing the non-sulfated *N-*glycan at the *N-*glycosylation site in the IgA tailpiece. **(A)** HCD.step spectrum shows B and Y ions confirming the sulfated *N-*glycan HexNAc_4_Hex_5_Fuc_1_NeuAc_2_Sulfo_1_. B and Y ions supporting structural evidence on sulfated *N-*glycans highlighted in yellow. **(B)** HCD.step spectrum shows the B and Y ions observed in the common non-sulfated *N-*glycan HexNAc_4_Hex_5_Fuc_1_NeuAc_2_. The annotation of the *N*-glycan composition enclosing the numbers in parentheses is required by the search engine.

As a result, the sulfated *N-*glycan, FA2G2S2-SO_4_, previously reported through glycomic analyses (Chuzel et al., 2021; Cajic et al., 2023; Burock et al., 2025), was identified in several gPSM exhibiting the tailpiece site N340-IgA1/N327-IgA2_m1/n_ and in a fewer gPSM presenting the C_H_2 domain site N205-IgA2. The validated gPSMs exhibiting sulfated *N-*glycans in both IgA samples are shown in Supplementary Table S3. Figure 2 shows b and y ion evidence of a peptide bearing a sulfated *N-*glycan at the tailpiece, compared to an identical glycopeptide without sulfation. Further evidence supporting the site-specific identification of the FA2G2S2-SO_4_ *N-*glycan was obtained through *de novo* sequencing of an HCD.low scan and is shown in Supplementary Figure S1. This Supplementary Figure shows the oxonium ion HexNAc_1_Hex_1_NeuAc_1_Sulfo_1_ [M+H]^+^ confirming sulfation at the terminal HexNAc, which agrees with the structure of the sulfated *N-*glycan characterized by Cajic et al., Chuzel et al., and Burock et al. via glycomic analyses (Chuzel et al., 2021; Cajic et al., 2023; Burock et al., 2025).

**FIGURE 3:**
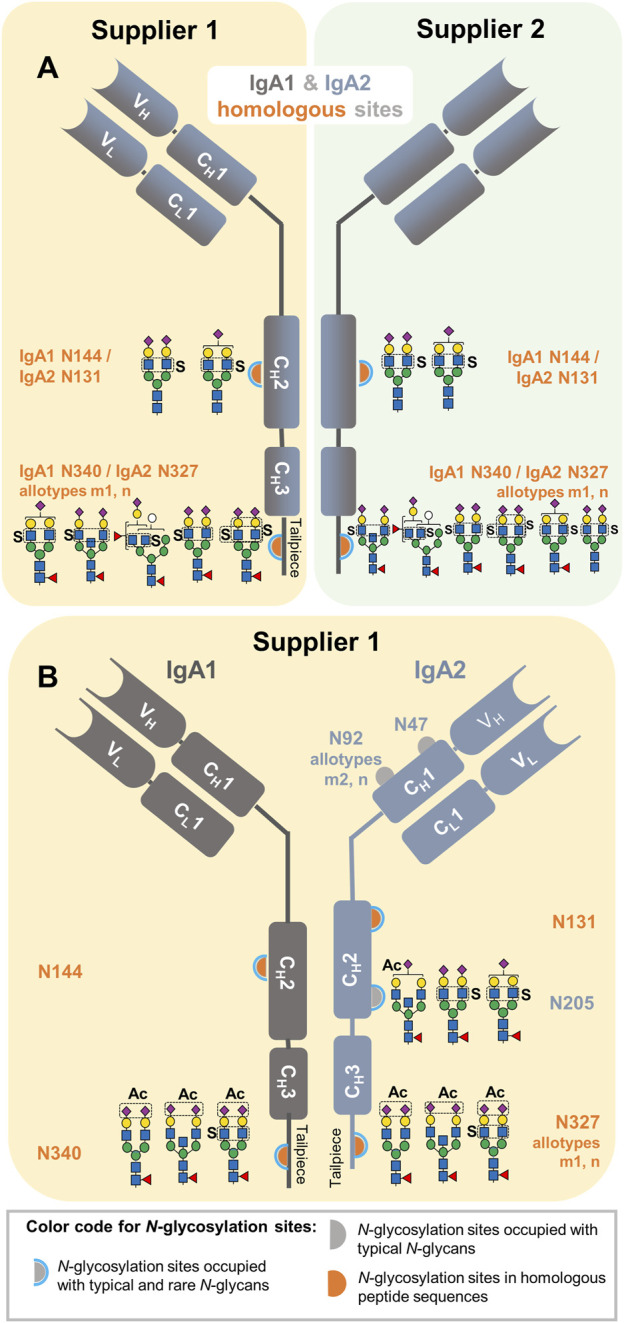
Site-specific description of the here identified *N-*glycans carrying HexNAc sulfation and NeuAc *O-*acetylation in commercially available human serum IgA samples from two suppliers. **(A)** Sulfated *N-*glycans identified in IgA1 and IgA2 homologous sites. **(B)** Sulfated *N*-glycans identified in unique *N*-glycosylation sites of IgA2 and NeuAc *O*-acetylated *N*-glycans identified within the entire analysis. The sample in which each *N*-glycan composition was identified is also indicated in yellow and green for the IgA sample from supplier 1 and 2, respectively.

In addition to the sulfated *N-*glycan HexNAc_4_Hex_5_Fuc_1_NeuAc_2_Sulfo_1,_ (reported as FA2G2S2-SO_4_ in our previous glycomic studies (Chuzel et al., 2021; Cajic et al., 2023; Burock et al., 2025), six new *N-*glycan compositions, for which sulfated-HexNAc oxonium marker ions were detected, could be identified using GlycoMod tool (Cooper et al., 2001) as described in the Materials and Methods section of this work. The structures of the new sulfated *N-*glycan compositions were described to some extent through their HCD.low and HCD.step fragment ion spectra. The Supplementary Figures S2–S7 present the manual annotation of these six new sulfated *N-*glycans. These spectra revealed one HexNAc-sulfated di-fucosylated hybrid-type *N-*glycan, two HexNAc-sulfated sialylated complex-type *N-*glycans without core fucosylation, one HexNAc-sulfated sialylated bisected complex-type *N-*glycan with core fucosylation, and one HexNAc-sulfated mono-sialylated complex-type *N-*glycan with core fucosylation. A sixth sialylated and core fucosylated complex-type *N-*glycan holding two sulfated monosaccharides (di-sulfated) was identified (Supplementary Figure S4); while sulfated HexNAc is evident, it was not possible to confirm the position of the second sulfation. As Figure 3 shows, our work not only describes a larger list of sulfated *N-*glycans but also provides a site-specific overview of IgA sulfated *N-*glycans for the first time. In this Figure, it is observed that the seven sulfated *N-*glycan compositions we detected were identified in the IgA samples from supplier 1 and supplier 2.

### 3.2 Site-specific identification of sialic acid *O-*acetylated *N-*glycans

As listed in the Supplementary Table S2, one *N-*glycan bearing *O-*acetylation of sialic acid (HexNAc_4_Hex_5_Fuc_1_NeuAc_2_Ac_1_) has been associated to IgA1 (Alagesan et al., 2019; GlyConnect, 2019a; GlyConnect, 2019b). First, MS^2^ scans were screened for NeuAc *O-*acetylated oxonium marker ions. Once their presence was confirmed, the strategies to maximize the identification of unknown *N-*glycan compositions with *O-*acetylated NeuAc were applied as described in Materials and Methods.

This process achieved not only the identification of the reported NeuAc *O-*acetylated *N-*glycan at the tailpiece site N340-IgA1/N327-IgA2_m1/n_ (Alagesan et al., 2019; GlyConnect, 2019a; GlyConnect, 2019b), but also three new NeuAc *O-*acetylated *N-*glycan compositions. As displayed in Figure 3B, all the NeuAc *O-*acetylated *N-*glycans identified here are of the complex-type, with and without bisecting GlcNAc, core-fucosylated and sialylated. Interestingly, NeuAc *O-*acetylated *N-*glycans were only identified in the human serum IgA sample from supplier 1 (Supplementary Table S3). All NeuAc *O-*acetylated *N-*glycans detected hereby were *de novo* sequenced to describe them in detail (Supplementary Figures S8–S11). One of the NeuAc *O-*acetylated *N-*glycans found at the tailpiece homologous site also bears sulfated HexNAc. An HCD.low MS^2^ spectrum acquired from this glycopeptide, not only presents oxonium ions from *O-*acetylated sialic acid, but also Hex_1_HexNAc_1_Sulfo_1_ [M+H]^+^ oxonium ion and Y ions demonstrating sulfation at the terminal HexNAc (Supplementary Figure S11A). Moreover, the HexNAc_1_Sulfo_1_ [M+H]^+^ oxonium marker ion was evident in the HCD.step MS^2^ spectrum (Supplementary Figure S11B). The parameters of the glycoproteomic search obtaining correct gPSMs presenting NeuAc *O-*acetylated oxonium marker ions were recorded for performing an expanded glycoproteomic search (Supplementary Table S1).

### 3.3 Peptide modifications detected in IgA *N-*glycopeptides

A key adjustment for the *N-*glycopeptide search was the peptide modifications. Multiple gPSMs with the tailpiece *N-*glycosylation site N340-IgA1/N327-IgA2_m1/n_ correspond to different mass variations of the predicted tryptic peptide. We found that these shifts in peptide mass were caused by one or two amino acid oxidations, one missed cleavage, or a truncated tailpiece form in which the C-terminal tyrosine is absent—this tyrosine truncated tailpiece form has been previously reported (Gomes et al., 2008; Klapoetke et al., 2011; Bondt et al., 2016). Interestingly, several gPSM containing the tailpiece site were identified by a semi-specific tryptic search (Supplementary Table S1), thus matching a C-terminal ragged peptide. One example is the peptide LAGKPTHVNVS. V lacking the last 11 amino acids of the tailpiece (VVMAEVDGTCY) (Supplementary Table S3). We observed that when setting only a tryptic search, this peptide matches an *N-*glycopeptide of the aldehyde dehydrogenase family 16 member A1 protein, but without showing evidence of b and y ions.

### 3.4 Identification of rare *N-*glycopeptides from “contaminant” proteins

The screening for MS^2^ scans containing rare oxonium ions was conducted using the list of oxonium marker ions reported by Zuniga-Banuelos et al. (2025). The oxonium ion Hex_1_HexNAc_1_HexA_1_ [M+H]^+^, (m/z 542.1716) was detected in the serum IgA samples from both suppliers. This oxonium ion serves as an indicator of *N-*glycans carrying human natural killer-1 (HNK-1) glycoepitope, which is present in human brain *N-*glycans and bears sulfated glucuronic acid (Helm et al., 2022). Therefore, it is assumed that the type of hexuronic acid observed in our study is glucuronic acid (Yamada et al., 2020; Helm et al., 2022). Following the strategies to maximize the identification of unknown *N-*glycan compositions (described in Materials and Methods), two *N-*glycan compositions were identified, one of which also contained sulfation as a modification. Then, the *N-*glycoproteomic search revealed that these *N-*glycans do not belong to any *N-*glycosylation site of IgA1 or IgA2 but to α1-antitrypsin (N271) and α-2-HS-glycoprotein (N156) (Supplementary Table S3). To elucidate their *N-*glycan structure, the *N-*glycopeptides were *de novo* sequenced (Supplementary Figures S12, S13). Although both *N-*glycopeptides carry di-antennary complex-type *N-*glycans with one sialic acid and one glucuronic acid as capping sugars, the α-2-HS-glycoprotein *N-*glycopeptide showed the oxonium ion Hex_1_HexNAc_1_HexA_1_Sulfo_1_ [M+H]^+^, thereby confirming the presence of the sulfated glycoepitope HNK-1.

HexNAc-sulfated oxonium marker ions were also detected in gPSMs of other proteins (Supplementary Table S3), such as Immunoglobulin J chain bearing HexNAc_4_Hex_5_NeuAc_2_Sulfo_1_ *N-*glycan at N71 site. The sulfated *N-*glycan HexNAc_4_Hex_5_Fuc_1_NeuAc_2_Sulfo_1_ was also found on *N-*glycopeptides from α-2-HS-glycoprotein (N156) and Complement C3 (N85), although their gPSMs present a low number of b and y ions.

### 3.5 Evaluation of the relative abundance of each IgA subclass

As described in Materials and Methods, we calculated the relative abundance of each IgA subclass based on the quantification of selected peptides identified by Proteome Discoverer. The peptide identification list is provided in Supplementary Table S4. Around 80% of the protein sequence of IgA1 and IgA2 is homologous. Therefore, some peptides are assigned to both IgA subclasses, which can produce misleading results. In order to avoid bias caused by tryptic peptides with homologous IgA sequences (ambiguous peptides), the non-glycopeptides “NFPPSQDASGDLYTTSSQLTLPATQCLAGK” and “NFPPSQDASGDLYTTSSQLTLPATQCPDGK” were selected to represent IgA1 and IgA2, respectively. We found that the ratio between IgA1:IgA2 subclasses was 89:11 in the commercial sample from supplier 1, which is the natural ratio in blood serum (Kerr, 1990), and approximately 99:1 in the one from supplier 2, which is ten times higher (Supplementary Table S5).

### 3.6 Expanded description of the micro-heterogeneity of IgA *N-*glycosylation

An expanded *N-*glycoproteomic search was conducted after obtaining new insights on sulfated, *O-*acetylated, and glucuronidated *N-*glycan compositions, as well as peptide modifications present in both IgA samples. To this end, a proteomic search plus multiple dedicated *N-*glycoproteomic searches featuring modified and common *N-*glycan compositions were conducted using Byonic™ as a search engine and implementing oxonium ions MS/MS filters specific to each *N-*glycan modification (Supplementary Table S1). In order to include (*N-*glyco)peptides from “contaminant” proteins, the human canonical proteome UniProtKB was set as protein database for the proteomic search, and a focused database was extracted from each sample based on the proteins identified. As described in Materials and Methods, (*N-*glyco)peptide identifications with a “Byonic MS2 search score” below 100 were excluded due to their poor quality and the remaining *N-*glycopeptide identifications were manually validated. During validation, gPSM presenting rare *N-*glycans were accepted as “True” if their corresponding oxonium marker ions were present in the MS^2^ spectra. Supplementary Table S3 shows peptides and *N-*glycopeptides identified among all replicates from both samples, belonging to IgA subclasses and “contaminant” proteins. The majority of “contaminant” *N-*glycopeptides belong to α1-antitrypsin, complement C3, kinninogen-1, and α-2-HS-glycoprotein. A site-specific relative quantification of the IgA *N-*glycosylation per sample was conducted. Considering that ambiguous peptides represent the IgA tailpiece site (N340-IgA1/N327-IgA2_m1/n_) and the C_H_2 domain site (N144-IgA1/N131-IgA2), the micro-heterogeneity analysis on those homologous *N-*glycosylation sites is assigned to both IgA subclasses (Supplementary Tables S6, S7). The micro-heterogeneity analysis for unique *N-*glycosylation sites of IgA2 for each sample is listed separately in Supplementary Tables S8, S9.

**FIGURE 4:**
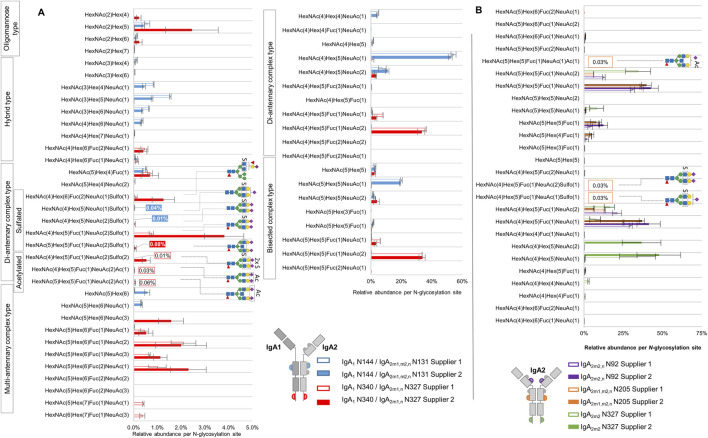
Comparison of micro-heterogeneity of *N-*glycosylation identified in commercially available human serum IgA samples from two suppliers. **(A)** Relative abundance of *N-*glycans at the homologous *N-*glycosylation sites N340-IgA1/N327-IgA2_(m1/n)_ and N144-IgA1/N131-IgA2. **(B)** Relative abundance of *N-*glycans at the *N-*glycosylation sites that are exclusively present in IgA2 subclasses. The color and format of the labels with values agree with the IgA sample supplier color code indicated in the legend. Results for relative quantification are shown in the Supplementary Tables S6–S9.

We first wanted to confirm that our *N-*glycoproteomic analysis reflects the typical high abundance *N-*glycans reported for each *N-*glycosylation site of the human serum IgA subclasses (Bondt et al., 2016; Plomp et al., 2018; Chandler et al., 2019; Clerc et al., 2023). Demonstrating the consistency of our analysis with other works, the bar plot in Figure 4A displays that the tailpiece site N340-IgA1/N327-IgA2_m1/n_ harbors core fucosylated sialylated complex-type di- and multi-antennary *N-*glycans, while the C_H_2 domain site N144-IgA1/N131-IgA2 bears mostly non-fucosylated sialylated hybrid- and di-antennary complex-type *N-*glycans. Also, in agreement with a preceding study, Figure 4B shows that the *N-*glycosylation sites that belong only to IgA2 subclass (N92-IgA2_m2/n_ and N205-IgA2) predominantly show sialylated core-fucosylated complex-type *N-*glycans, including bisecting *N-*glycans (Chandler et al., 2019). We only observed one *N-*glycopeptide representing the N47-IgA2 site with a mono-sialylated core-fucosylated complex-type *N-*glycan and a few *N-*glycopeptides presenting complex-type *N-*glycans at the N327-IgA2_m2_ (Supplementary Tables S8, S9).

In regard to NeuAc *O-*acetylated *N-*glycans, the analysis reflected that these *N-*glycans are very low abundant. Nevertheless, three of the four *O-*acetylated *N-*glycans we identified were picked by Byonic™ in at least three of the four technical replicates of IgA sample from supplier 1, and their relative abundance was estimated here for the first time. As Figure 4A shows, at the tailpiece site N340-IgA1/N327-IgA2_m1/n_, two NeuAc *O-*acetylated *N-*glycans display a total relative abundance of 0.09% (Supplementary Table S6). At the C_H_2 domain site N205-IgA2 (Figure 4B), a mono-sialylated bisected NeuAc *O-*acetylated complex-type *N-*glycan was observed with an average relative abundance of 0.03% (Supplementary Table S8). The *O-*acetylated complex-type *N-*glycan bearing both, NeuAc *O-*acetylation and HexNAc sulfation, appeared only in one technical replicate of the sample from supplier 1, hence, it was not part of the quantification.

Figure 4 displays that the sulfated *N-*glycan HexNAc_4_Hex_5_Fuc_1_NeuAc_2_Sulfo_1_ (previously reported by us via glycomic analyses as FA2G2S2-SO_4_ (Chuzel et al., 2021; Cajic et al., 2023; Burock et al., 2025) was identified with the highest abundance at the tailpiece *N-*glycosylation site N340-IgA1/N327-IgA2_m1/n_ (0.74% and 3.85% in the sample from supplier 1 and supplier 2, respectively). In contrast, the same *N-*glycan was identified in a very low abundance at the C_H_2 domain site N205-IgA2 (only 0.03% of the total *N-*glycans in the sample from supplier 1, Supplementary Table S8). At the C_H_2 domain site N144-IgA1/N131-IgA2, the samples from both suppliers bear non-fucosylated sialylated sulfated di-antennary complex-type *N-*glycans in very low relative abundance. In fact, only the sample from supplier 2 returned an area under the curve value for at least three technical replicates, summing a total relative abundance of 0.05% of sulfated *N-*glycans at the C_H_2 domain homologous site (Supplementary Table S7). The di-sulfated complex-type *N-*glycan HexNAc_4_Hex_5_Fuc_1_NeuAc_2_Sulfo_2_, detected at the tailpiece site, shows 0.01% and 0.54% in the sample from supplier 1 and supplier 2, respectively. Overall, while the IgA sample from supplier 2 shows the highest relative abundance of sulfated *N-*glycans at the IgA1/IgA2 homologous sites, only the sample from supplier 1 presents peak area values of sulfated *N-*glycans that belong only to IgA2 *N-*glycosylation sites.

### 3.7 Comparison of two data analysis approaches for the identification of sulfated *N-*glycopeptides

**FIGURE 5:**
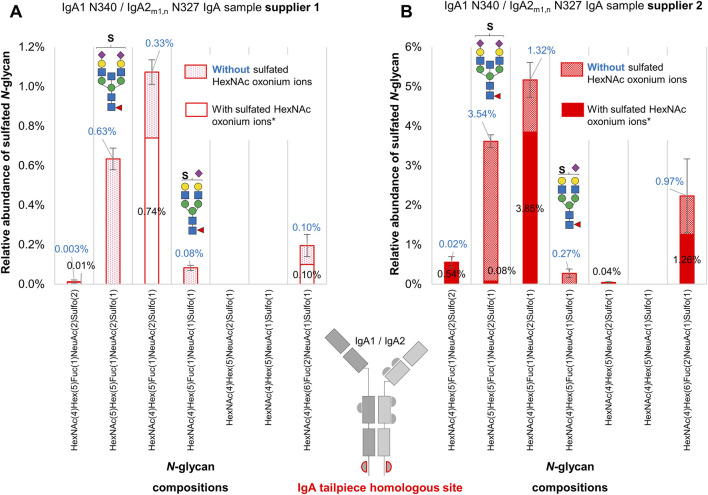
A comparative analysis of sulfated *N-*glycans with and without HexNAc-sulfated oxonium ions attached to the IgA tailpiece site in commercially available human serum IgA samples from two suppliers. **(A)** The sample from supplier 1. **(B)** The sample from supplier 2. Sulfated *N-*glycopeptides were identified, manually validated, and verified for oxonium marker ions through Byonic™. Two sulfated *N-*glycan structures, one with bisecting HexNAc and another mono-sialylated, appear more abundant in glycopeptide spectra matches when their spectra do not present oxonium marker ions of sulfated HexNAc. Asterisk (*) indicates that oxonium-ion-based evidence refers to detection of Sulfo_1_HexNAc_1_ [M+H]^+^ or Sulfo_1_HexNAc_1_Hex_1_ [M+H]^+^ oxonium ions.

To address the challenge of identifying oxonium fragment ions derived from sulfated *N-*glycans, a second set of *N-*glycopeptide searches without applying sulfated oxonium ions as part of the MS/MS filter was conducted and manually validated. The sulfated *N-*glycopeptides identified included sulfated gPSM that exhibited the presence or absence of HexNAc-sulfated oxonium ions in the MS^2^ spectra (Supplementary Table S10). During manual validation, the sulfated gPSM lacking HexNAc-sulfated oxonium ions were assigned as “True” if the match was correct regarding the b and y fragment ions, the conserved peptide fragmentation pattern, and common glycan oxonium ions [e.g., HexNAc_1_ (M+H)^+^, NeuAc_1_ (M+H)^+^, HexNAc_1_Hex_1_ (M+H)^+^]. Then, gPSM bearing sulfated *N-*glycans were classified by the presence or absence of HexNAc-sulfated oxonium marker ions. As the homologous tailpiece site (N340-IgA1/N327-IgA2_m1/n_) is the primary source of sulfated *N-*glycans, the relative quantification of sulfated *N-*glycans focused exclusively on this site (Supplementary Table S11). The abundance of each *N-*glycan composition was normalized by the total area under the curve of all *N-*glycan compositions identified at the homologous tailpiece site and is displayed in Figure 5.

As can be observed in Figure 5, there is an increase of approximately 30% in the relative abundance of the composition HexNAc_4_Hex_5_Fuc_1_NeuAc_2_Sulfo_1_ (related to the reported *N-*glycan FA2G2S2-SO_4_ (Chuzel et al., 2021; Cajic et al., 2023; Burock et al., 2025) in both samples. One of the biggest differences between the sulfated *N-*glycopeptide identified within each glycoproteomic search is the presence of the sulfated bisected *N-*glycan HexNAc_5_Hex_5_Fuc_1_NeuAc_2_Sulfo_1_. With the relative quantification of sulfated *N-*glycopeptides presenting HexNAc-sulfated oxonium ions, the sulfated bisected *N-*glycan only appears in one of the replicates of the sample from supplier 1, and with very low relative abundance (0.08%) in the sample from supplier 2. However, by considering sulfated *N-*glycopeptides lacking HexNAc-sulfated oxonium ions, this bisected *N-*glycan becomes the second most predominant sulfated *N-*glycan in both samples, reflecting an increase of 0.63% and 3.54% in the samples from supplier 1 and supplier 2, respectively. Although the *N-*glycopeptide identifications bearing the sulfated bisected *N-*glycan lacking HexNAc-sulfated oxonium ions are detected within independent chromatographic peaks, the average scan time of the precursor ions representing those peaks is not significantly different from the identifications presenting HexNAc-sulfated oxonium ions. The case is similar for the sulfated mono-sialylated *N-*glycan, whose relative abundance becomes evident by including sulfated *N-*glycopeptides lacking HexNAc-sulfated oxonium ions.

These results point to a broader picture. On the one hand, it is possible that these differences in sulfated *N-*glycopeptide abundances are caused by the instability of the HexNAc-sulfated oxonium ions after fragmentation, generating MS^2^ spectra lacking these ions. On the other hand, it is possible that different sulfation modifications coexist in the IgA sample, like galactose sulfated *N-*glycans, which has also been reported in human IgA via glycomic analyses (Alagesan et al., 2019; GlyConnect, 2019a; GlyConnect, 2019b).

## 4 Discussion

Our investigation includes, for the first time, HexNAc-sulfated and NeuAc *O-*acetylated *N-*glycans in the micro-heterogeneity description of IgA *N-*glycosylation. GlyConnect *N-*glycan databases corresponding to IgA1 and IgA2_m2_ (UniProt entries: P01876 and P01877, respectively) were screened for sulfated and NeuAc *O*-acetylated *N-*glycan compositions (Alocci et al., 2019; Expasy, 2019). In regard to *N-*glycans bearing NeuAc *O-*acetylation, our work can contribute to adding three new *N-*glycan compositions to the database, even in a site-specific manner. GlyConnect IgA1 and IgA2 *N-*glycan databases showed only three of the seven sulfated *N-*glycan compositions detected by us: HexNAc_4_Hex_5_Fuc_1_NeuAc_2_Sulfo_1_, HexNAc_4_Hex_5_Fuc_1_NeuAc_1_Sulfo_1_, and HexNAc_4_Hex_5_NeuAc_2_Sulfo_1_ (Supplementary Table S2) (Alagesan et al., 2019; GlyConnect, 2019a; GlyConnect, 2019b). However, these three *N-*glycans were reported to be modified with galactose sulfation instead of GlcNAc sulfation. Alagesan et al. conducted an advanced derivatization approach including permethylation (Alagesan et al., 2019). Permethylation is known to reduce the suppression of sulfated *N-*glycan precursor ions, as it neutralizes the signals of sialylated *N-*glycans (Khoo and Yu, 2010), which are abundant *N-*glycans in IgA. These highly optimized derivatization steps might have favored the detection of galactose-sulfated *N-*glycans regardless of their lower abundance. Typically, permethylation derivatization and negative ion mode LC-MS/MS analysis are gold standard techniques for the characterization of negatively charged *N-*glycans (Cheng et al., 2015; Kuo and Khoo, 2020). However, all acquisitions in the present study were carried out in positive ion mode, guided by the following considerations. First, acquisition using LC-MS/MS in positive ion mode with HCD is a well-established method (Hoffmann et al., 2018), that has demonstrated its effectiveness in identifying peptides bearing sulfated, glucuronidated, and other rare *N-*glycans in human blood plasma proteins (Zuniga-Banuelos et al., 2025). Second, other studies also confirmed the reliable detection of sulfated oxonium ions using LC-MS/MS in positive ion mode with HCD as fragmentation method (Kuo et al., 2018; She et al., 2019). Third, by addressing the methodology described in valuable investigations on common IgA *N-*glycopeptides, it was confirmed that MS/MS in positive ion mode is the most widely applied technique (Gomes et al., 2008; Bondt et al., 2016; Plomp et al., 2018; Chandler et al., 2019; Clerc et al., 2023). Thus, the aim of our work was to expand the site-specific identification of IgA *N-*glycans to include rare *N-*glycans without excluding typically detected *N-*glycans within the evaluation of the site-specific IgA glycoprofile. We believe that using a widely applied technique for acquiring *N-*glycopeptide data is advantageous as it could be readily reproduced and adapted by other laboratories.

Our data analysis does not provide any probative structural information on galactose sulfation for two reasons. On the one hand, the oxonium marker ion Hex_1_Sulfo_1_ [M+H]^+^, expected from a sulfated galactose, was never detected in any of our gPSMs containing sulfated *N-*glycans. Interestingly, although we cannot confirm oxonium ions specific to galactose-sulfation, it cannot be dismissed the hypothesis that *N-*glycopeptide precursor ions bearing galactose-sulfated *N-*glycans were still fragmented. This hypothesis is supported by the work of Kuo et al., who used a highly refined glycopeptide enrichment approach to analyze sulfated *N-*glycopeptides derived from thyroglobulin from bovine thyroid (Kuo et al., 2018). This protein bears galactose-sulfated *N-*glycans (Spiro and Bhoyroo, 1988). They employed a very comparable LC-MS/MS positive ion mode method and compared three fragmentation regimes, including HCD technique. Kuo et al. found that it was possible to detect the sulfated LacNAc oxonium ion in a very low intensity. However, none of their techniques detected the galactose sulfated oxonium ion. This indicates that detecting galactose-sulfated oxonium ion from underivatized galactose-sulfated *N-*glycans using LC-MS/MS positive ion mode is still a difficult task. On the other hand, as reported by She et al., the detection of galactose sulfation in intact *N-*glycopeptides relies on the manual identification of Y ions holding terminal galactose sulfation (She et al., 2019). Although manual validation can pursue the identification of Y ions holding terminal galactose sulfation, software tools capable of comprehensively matching Y ions can facilitate this identification process. Therefore, the identification of *N-*glycopeptides bearing galactose sulfated *N-*glycans still requires establishing a reliable and less time-consuming computational strategy for the identification of the expected Y ions (She et al., 2019). For example, GlycanFinder software performs *de novo* sequencing of *N-*glycopeptides and can be customized to identify peptides bearing galactose-sulfated *N-*glycans (Sun et al., 2023).

Overall, both samples consistently showed that the primary source of HexNAc-sulfated *N-*glycans in IgA is the tailpiece *N-*glycosylation site. It was also observed that the relative abundance of NeuAc *O-*acetylated *N-*glycans at the tailpiece *N-*glycosylation site is around ten times lower than the relative abundance of sulfated *N-*glycans within the sample from supplier 1. Interestingly, *O-*acetylated *N-*glycans were not detected in the sample from supplier 2, which exhibited a massive depletion of IgA2 subclass—present within an IgA1:IgA2 ratio ten times lower than the normal ratio in human serum (Kerr, 1990). This ratio can be affected by the purification process applied (Reinhart and Kunert, 2015). Differences in *N-*glycosylation between both IgA subclasses have been demonstrated in preceding studies. By separation of IgA1 and IgA2 subclasses, Steffen et al. have described that the total IgA2 *N-*glycome shows low levels of sialylated bisected complex-type *N-*glycans and higher levels of hybrid- and oligomannose-type *N-*glycans (Steffen et al., 2020). Chandler et al. also showed, by the specific glycoproteomic analysis of IgA2, that the C_H_2 domain site N131-IgA2 bears hybrid-type *N-*glycans in higher abundance than the C_H_2 domain site N144 in IgA1 (Chandler et al., 2019). These studies suggest that the share of hybrid-type *N-*glycans will be influenced by the IgA1:IgA2 ratio, which might explain the differences observed in our analysis between both commercial samples (Figure 4A). A question that also arises is to what extent the IgA1:IgA2 subclass ratio affects the abundance of *O-*acetylated and sulfated *N-*glycans. We hypothesize that the differences in the relative abundance of sulfated *N-*glycopeptides detected in our analyses within both IgA samples might be related to their IgA1:IgA2 ratio. Interestingly, this would suggest that sulfated *N-*glycans are selectively more conserved at the tailpiece *N-*glycosylation site in IgA1 and not in IgA2. Nonetheless, further analyses are required to elucidate differences in the dominance of sulfated *N-*glycans between the IgA subclasses.

New methods have been developed for assisting the detailed analysis of sulfated and *O-*acetylated *N-*glycans. Recently, Cajic et al. established and applied a workflow for multiple analyses of special *N-*glycans by means of removable fluorescent labeling (Fmoc) (Cajic et al., 2023). Using this method, they isolated and exhaustively characterized *O-*acetylated *N-*glycans from horse serum proteins. Another application of this method, was the isolation of a sulfated *N-*glycan from Fmoc-labeled *N-*glycans released from human serum IgA, for further analysis via both MALDI-TOF and xCGE-LIF. The xCGE-LIF glycomic analysis was combined with a highly specific sulfatase and a sulfate-dependent hexosaminidase, both characterized by Chuzel et al. (2021). Also, Chuzel et al. found that this sulfatase can act as a lectin, which is highly selective for GlcNAc-6-SO_4_ in absence of calcium. Even though each methodology has limitations, cutting-edge glycoanalytical tools can complement the structural elucidation of these rare *N-*glycans.

Our glycoproteomic workflow demonstrates the importance of considering the contribution of *N-*glycans from “contaminant” proteins. We detected contaminant proteins and rare *N-*glycans bearing the HNK-1 glycoepitope in a few of these contaminant proteins. The presence of these “contaminant” proteins in the purified IgA is probably due to chromatographic co-elution or to stable protein-protein interactions during the purification process (Doi et al., 1984; Martinez-Flores et al., 2015; Reinhart and Kunert, 2015). Studies show that proteins like complement C3 and β-2-glycoprotein form circulating immune complexes with IgA subclasses in blood serum (Doi et al., 1984; Martinez-Flores et al., 2015). Additionally, a strong interaction is controlled by the penultimate cysteine of the IgA tailpiece, forming a disulfide bond either with albumin or α1-antitrypsin (Vaerman et al., 1987). In this regard, our work proved that several gPSMs bearing typical and rare *N-*glycans within both IgA samples are represented by semi-tryptic tailpiece peptides, which have lost a significant number of amino acids (including the penultimate cysteine) from the C-terminal end. Studies have demonstrated that the penultimate cysteine (Cys352) is critical for IgA dimerization, and disulfide bond formation with J-chain and other proteins (Vaerman et al., 1987; Bastian et al., 1992; Atkin et al., 1996). Clarifying whether the shorter tailpiece peptides result from an unspecific cleavage or are another truncated tailpiece variant of the IgA subclasses requires further investigation.

It is not known how sulfated and NeuAc *O-*acetylated *N-*glycans influence the effector function of IgA subclasses. On the one hand, sulfated glycoepitopes, are ligands for L-selectin and this interaction plays an important role in cell adhesion and trafficking of immune system cells (Hemmerich et al., 1994; Mowery et al., 2004; Otsuki et al., 2010; Muthana et al., 2012). On the other hand, studies using glycan arrays demonstrate the affinity of specific influenza viruses (and also bacteria) for sialylated glycoepitopes containing sulfated GlcNAc (Scharfman et al., 2000; Liao et al., 2010; Wang et al., 2012). Also, NeuAc *O-*acetylation influences the binding to viral neuraminidases during infection (Rogers et al., 1986), and may have immunoregulatory effects by affecting the affinity of the sialylated glycan for lectins (e.g., CD22) or bacterial sialidases (Sjoberg et al., 1994; Weiman et al., 2009). It has also been reported that sialylated *N-*glycans at the IgA tailpiece play a role in the neutralization of influenza virus infection (Maurer et al., 2018). Therefore, it is possible that IgA sulfated and *O*-acetylated *N-*glycans also play a key role in the IgA antiviral activity, for instance, by mimicking a cell receptor-ligand used during infection.

## 5 Conclusion

Currently, the difficulty of detecting sulfated *N-*glycans by MS-based glycoproteomic approaches limits the site-specific elucidation of sulfated *N-*glycans attached to proteins, e.g., to IgA subclasses. IgA has clinical relevance due to its multiple roles in the immune system. In this work, we applied our previously developed in-depth *N-*glycoproteomic workflow (Zuniga-Banuelos et al., 2025) to study sulfated and other rare *N-*glycans on two commercially available human IgA samples isolated from blood serum. With this, we achieved the identification of the *N-*glycosylation sites harboring the sulfated *N-*glycan HexNAc_4_Hex_5_Fuc_1_NeuAc_2_Sulfo_1_ (FA2G2S2-SO_4_), reported up to now only by site unspecific *N-*glycomic investigations (Chuzel et al., 2021; Cajic et al., 2023; Burock et al., 2025). We detected new hybrid-type and complex-type HexNAc-sulfated *N-*glycans in IgA, which were overlooked in previous glycomic and glycoproteomic analyses (Gomes et al., 2008; Bondt et al., 2016; Plomp et al., 2018; Chandler et al., 2019; Steffen et al., 2020; Chuzel et al., 2021; Cajic et al., 2023; Clerc et al., 2023). However, we could not detect previously reported galactose-sulfated *N-*glycans in IgA (Alagesan et al., 2019; GlyConnect, 2019a; GlyConnect, 2019b). Also, we identified new *N-*glycan compositions holding *O-*acetylated NeuAc in a site-specific manner within IgA and glucuronidated *N-*glycans in contaminant proteins. Finally, we estimated the relative abundance of these sulfated and *O-*acetylated *N-*glycans per glycosylation site and IgA sample. Our data demonstrates for the first time, that the primary protein position for sulfated and *O-*acetylated *N-*glycans is the tailpiece of IgA. Our MS-based *N-*glycoproteomic workflow allows the investigation of very low abundant *N-*glycopeptide forms, like HexNAc-sulfated and NeuAc *O-*acetylated *N-*glycans. This workflow can be applied to other proteins (e.g., glycoproteins isolated from different body fluids, such as urine, milk, saliva, or mucosal secretions), in order to expand the overview of human glycobiology diversity. It is anticipated that our workflow supports the future site-specific evaluation of sulfated and *O-*acetylated *N-*glycans in IgA produced as a therapeutic or in clinical diagnostics.

## Data Availability

The data produced by the *N-*glycoproteomic and proteomic analyses here conducted is available within the supplemental data of this article. The MS raw files were deposited to the ProteomeXChange Consortium, identifier PXD060281, through MassIVE (dataset identifier MSV000096980); doi:10.25345/C5MP4W084; URL: https://massive.ucsd.edu/ProteoSAFe/dataset.jsp?task=0564f1d2b40b448caf36d6e7490ae6f5.

## Abbreviations

IgA, Human immunoglobulin A; S, Sulfation; Ac, *O*-Acetylation; NeuAc, *N*-acetylneuraminic acid; HexNAc, *N*-acetylhexosamine; Hex, Hexose; gPSM, glycopeptide spectra match; HCD.step, higher-energy collisional dissociation with stepped normalized collisional energy [NCE of 20, 35, and 50]; HCD.low, higher-energy collisional dissociation with fixed NCE of 20.
