# Supplementary material for: Immunoglobulin A carries sulfated and O-acetylated N-glycans primarily at the tailpiece site – strategies for site-specific N-glycan identification
Source: Front Mol Biosci. 2025 Aug 1;12:1595173. doi: 10.3389/fmolb.2025.1595173 (PMC12353717; doi:10.3389/fmolb.2025.1595173)
Supplement: Supplementary file 1 [file Supplementaryfile1.pdf]

List of Supplementary Figures

Supplementary Figure 1. Annotated data of a glycopeptide bearing the sulfated *N*-glycan HexNAc(4)Hex(5)Fuc(1)NeuAc(2)Sulfo(1).

Supplementary Figure 2. Annotated data of a glycopeptide bearing the sulfated *N*-glycan HexNAc(4)Hex(5)NeuAc(1)Sulfo(1).

Supplementary Figure 3. Annotated data of a glycopeptide bearing the sulfated *N*-glycan HexNAc(4)Hex(5)NeuAc(2)Sulfo(1).

Supplementary Figure 4. Annotated data of a glycopeptide bearing the sulfated N-glycan HexNAc(4)Hex(5)Fuc(1)NeuAc(2)Sulfo(2).

Supplementary Figure 5. Annotated data of a glycopeptide bearing the sulfated *N*-glycan HexNAc(5)Hex(5)Fuc(1)NeuAc(2)Sulfo(1).

Supplementary Figure 6. Annotated data of a glycopeptide bearing the sulfated *N*-glycan HexNAc(4)Hex(6)Fuc(2)NeuAc(1)Sulfo(1).

Supplementary Figure 7. Annotated data of a glycopeptide bearing the sulfated *N*-glycan HexNAc(4)Hex(6)Fuc(2)NeuAc(1)Sulfo(1).

Supplementary Figure 8. Annotated data of glycopeptide containing NeuAc *O*-acetylation in the *N*-glycan HexNAc(4)Hex(5)Fuc(1)NeuAc(2)Ac(1).

Supplementary Figure 9. Annotated data of glycopeptide containing the NeuAc *O*-acetylated *N*-glycan HexNAc(5)Hex(5)Fuc(1)NeuAc(1)Ac(1).

Supplementary Figure 10. Annotated data of glycopeptide containing NeuAc *O*-acetylation in the *N*-glycan HexNAc(5)Hex(5)Fuc(1)NeuAc(2)Ac(1).

Supplementary Figure 11. Annotated data of a glycopeptide containing NeuAc *O*-acetylation plus HexNAc sulfation in the *N*-glycan HexNAc(4)Hex(5)Fuc(1)NeuAc(2)Ac(1)Sulfo(1).

Supplementary Figure 12. Annotated data of a glycopeptide potentially containing HexA sulfation in the *N*-glycan HexNAc(4)Hex(5)NeuAc(1)HexA(1)Sulfo(1) of a contaminant protein.

Supplementary Figure 13. Annotated data of a glycopeptide potentially containing HexA sulfation in the *N*-glycan HexNAc(4)Hex(5)NeuAc(1)HexA(1) of a contaminant protein.

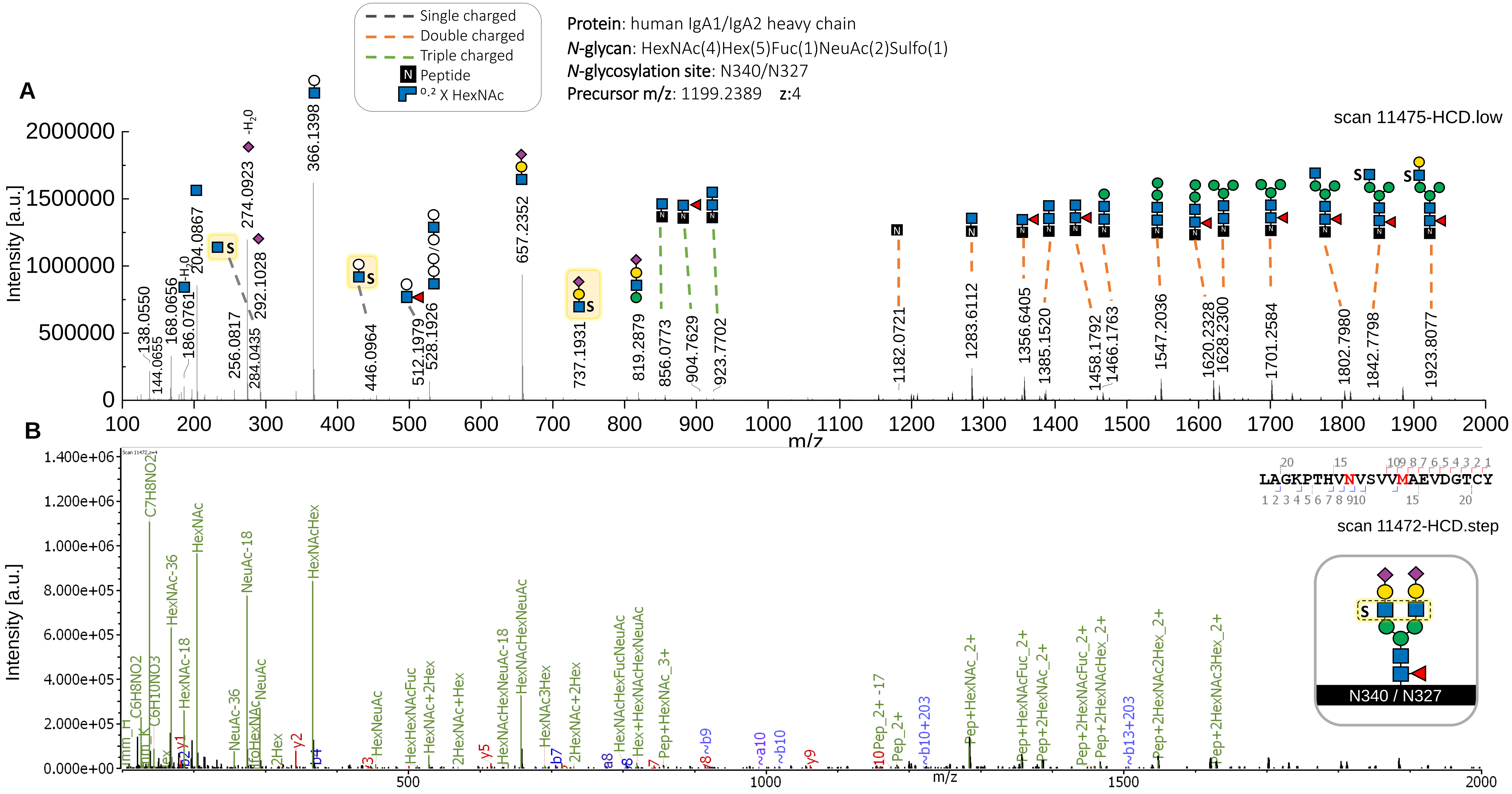

Supplementary Figure 1. Annotated data of a glycopeptide bearing the sulfated *N*-glycan HexNAc(4)Hex(5)Fuc(1)NeuAc(2)Sulfo(1). (A) Description of the *N*-glycan moiety through HCD.low spectrum shows B and Y ions. The oxonium marker ion $Sulfo_1HexNAc_1$/284.0435 $[M+H]^+$ confirms specifically HexNAc sulfation, while the oxonium ion $Sulfo_1HexNAc_1Hex_1NeuAc_1$/737.1931 $[M+H]^+$ confirms specifically antenna HexNAc sulfation. **(B)** The Byonic ™ annotation of the HCD.step spectrum refers to a tryptic peptide containing the homologous site N340-IgA1/N327-$IgA2_{(m1/n)}$. The HCD.step spectrum shows the oxonium marker ions confirming HexNAc sulfation. The HCD.low and HCD.step spectra that most extensively describe the glycoform and peptide sequence were selected for representing this glycopeptide.

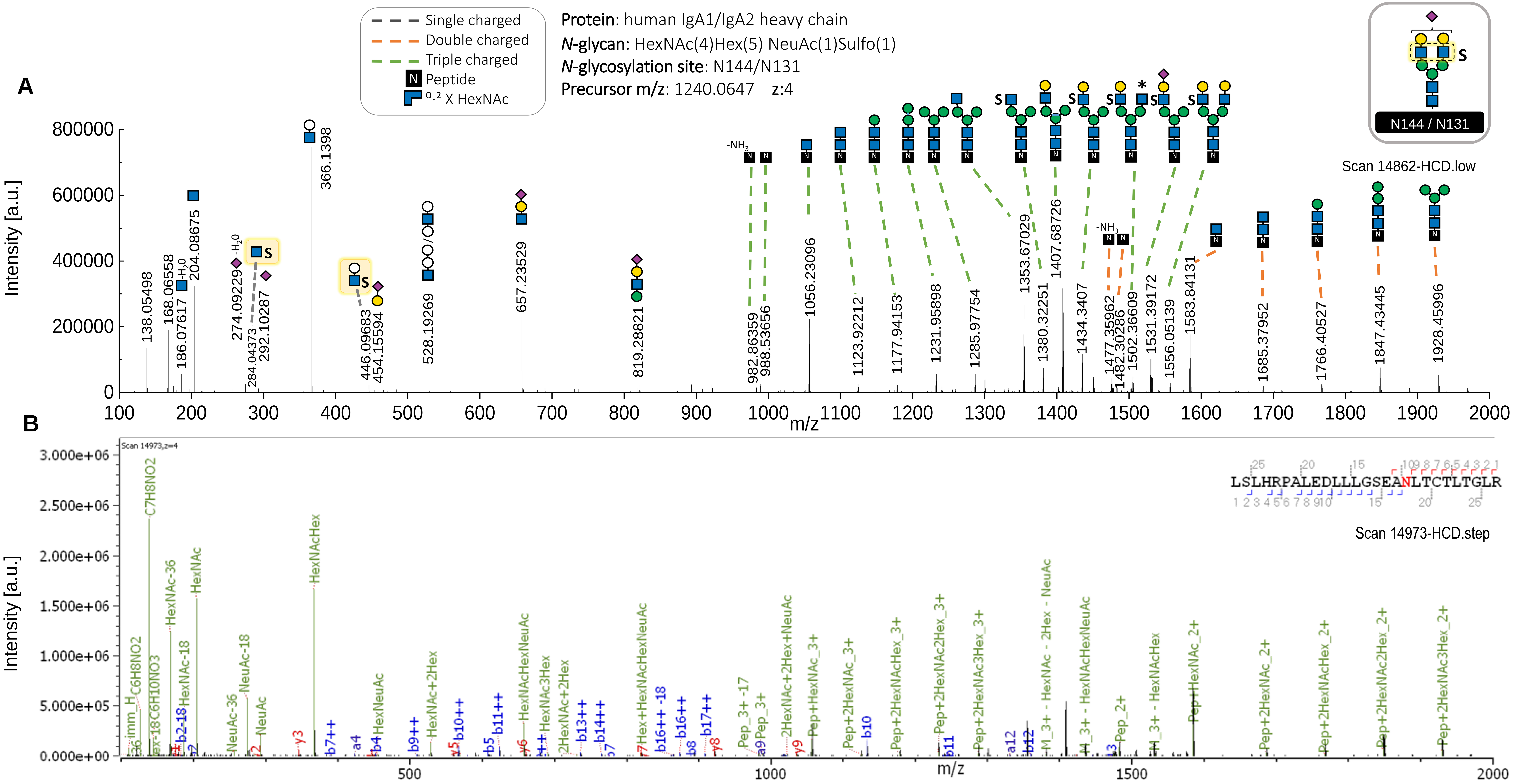

Supplementary Figure 2. Annotated data of a glycopeptide bearing the sulfated *N*-glycan HexNAc(4)Hex(5)NeuAc(1)Sulfo(1). (A) Description of the *N*-glycan moiety through HCD.low spectrum shows B and Y ions confirming antenna with a sulfated HexNAc. The *N*-glycan building blocks with an asterisk symbol (*) depict the *N*-glycan composition by a Y ion assigned to the second isotope peak of the expected m/z. **(B)** The Byonic ™ annotation of the HCD.step spectrum refers to an amino acid sequence of this *N*-glycopeptide containing the homologous site N144-IgA1/N131-IgA2$_{(m1/m2/n)}$. The HCD.step spectrum shows the oxonium marker ions confirming HexNAc sulfation ($Sulfo_1HexNAc_1$/284.0437 $[M+H]^+$). The HCD.low and HCD.step spectra that most extensively describe the glycoform and peptide sequence, acquired within the shortest time, were selected for representing this glycopeptide.

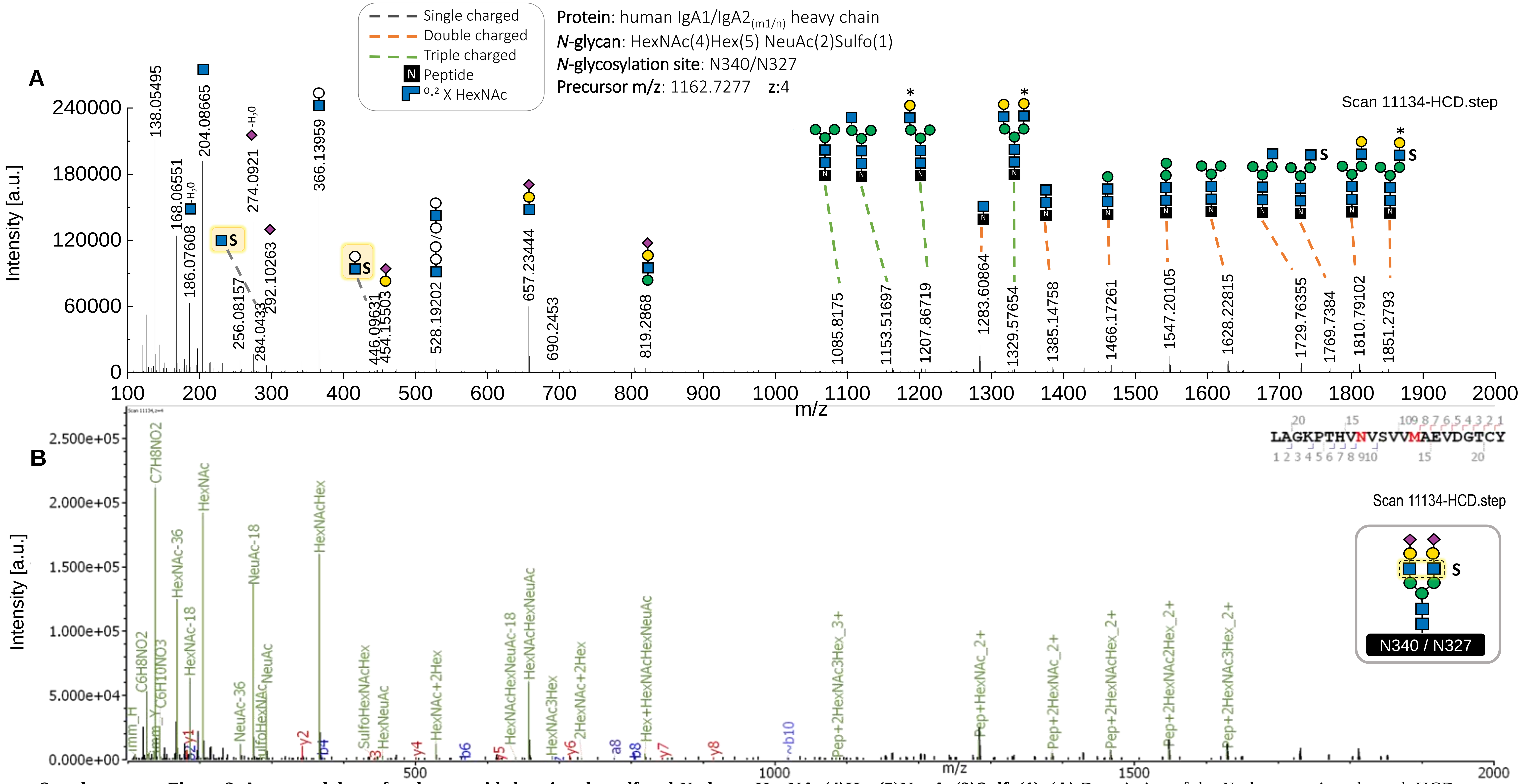

Supplementary Figure 3. Annotated data of a glycopeptide bearing the sulfated *N*-glycan HexNAc(4)Hex(5)NeuAc(2)Sulfo(1). (A) Description of the *N*-glycan moiety through HCD.step spectrum shows B and Y ions confirming antenna with sulfated HexNAc. The *N*-glycan building blocks with an asterisk symbol (*) depict the *N*-glycan composition by a Y ion assigned to the second isotope peak of the expected m/z. **(B)** Byonic ™ annotation of the HCD.step spectrum refers to an amino acid sequence of this *N*-glycopeptide containing the homologous site N340-IgA1/N327-IgA2$_{(m1/n)}$. The HCD.step spectrum shows the oxonium marker ions confirming HexNAc sulfation (Sulfo$_1$HexNAc$_1$/284.0433 $[M+H]^+$). The HCD.step spectrum that most extensively describes the glycoform and peptide sequence was selected for representing this glycopeptide.

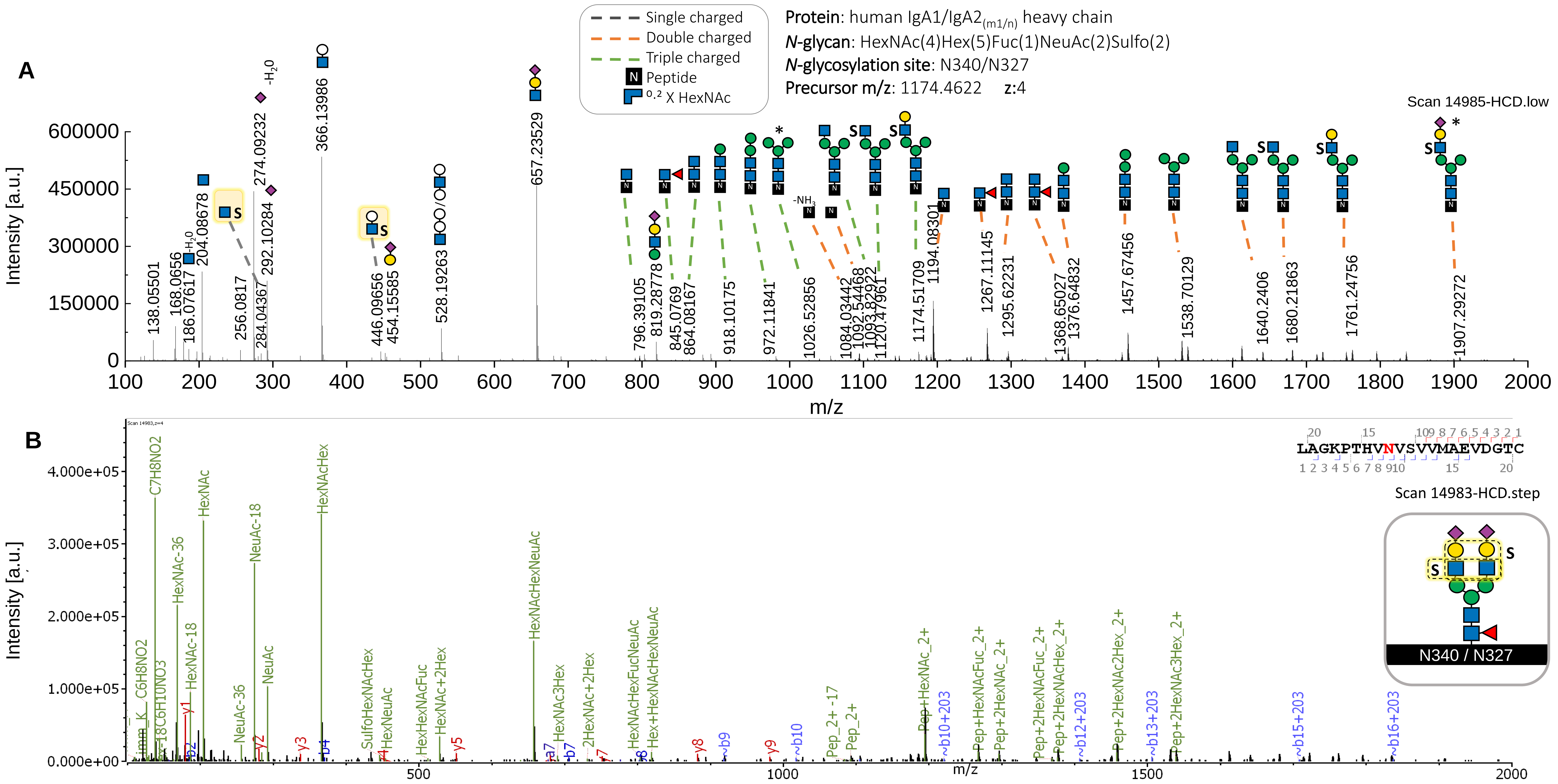

Supplementary Figure 4. Annotated data of a glycopeptide bearing the sulfated *N*-glycan HexNAc(4)Hex(5)Fuc(1)NeuAc(2)Sulfo(2). (A) Description of the *N*-glycan structure through HCD.low spectrum shows B and Y ions confirming one sulfation linked to antenna HexNAc, while the linkage of the second sulfation cannot be fully deduced and it can be associated to antenna HexNAc or Hex. The *N*-glycan building blocks with an asterisk symbol (*) continue to depict the *N*-glycan composition by a Y ion assigned to the second isotope peak of the expected m/z. **(B)** HCD.step spectrum annotated by Byonic ™ recognizes b and y ions corresponding to an amino acid sequence of this *N*-glycopeptide containing the homologous site N340-IgA1/N327-IgA2$_{(m1/n)}$. The HCD.step spectrum shows Sulfo$_1$HexNAc$_1$/284.0434 $[M+H]^+$ oxonium ion, confirming HexNAc sulfation. The HCD.low and HCD.step spectra that most extensively describe the glycoform and peptide sequence were selected for representing this glycopeptide.

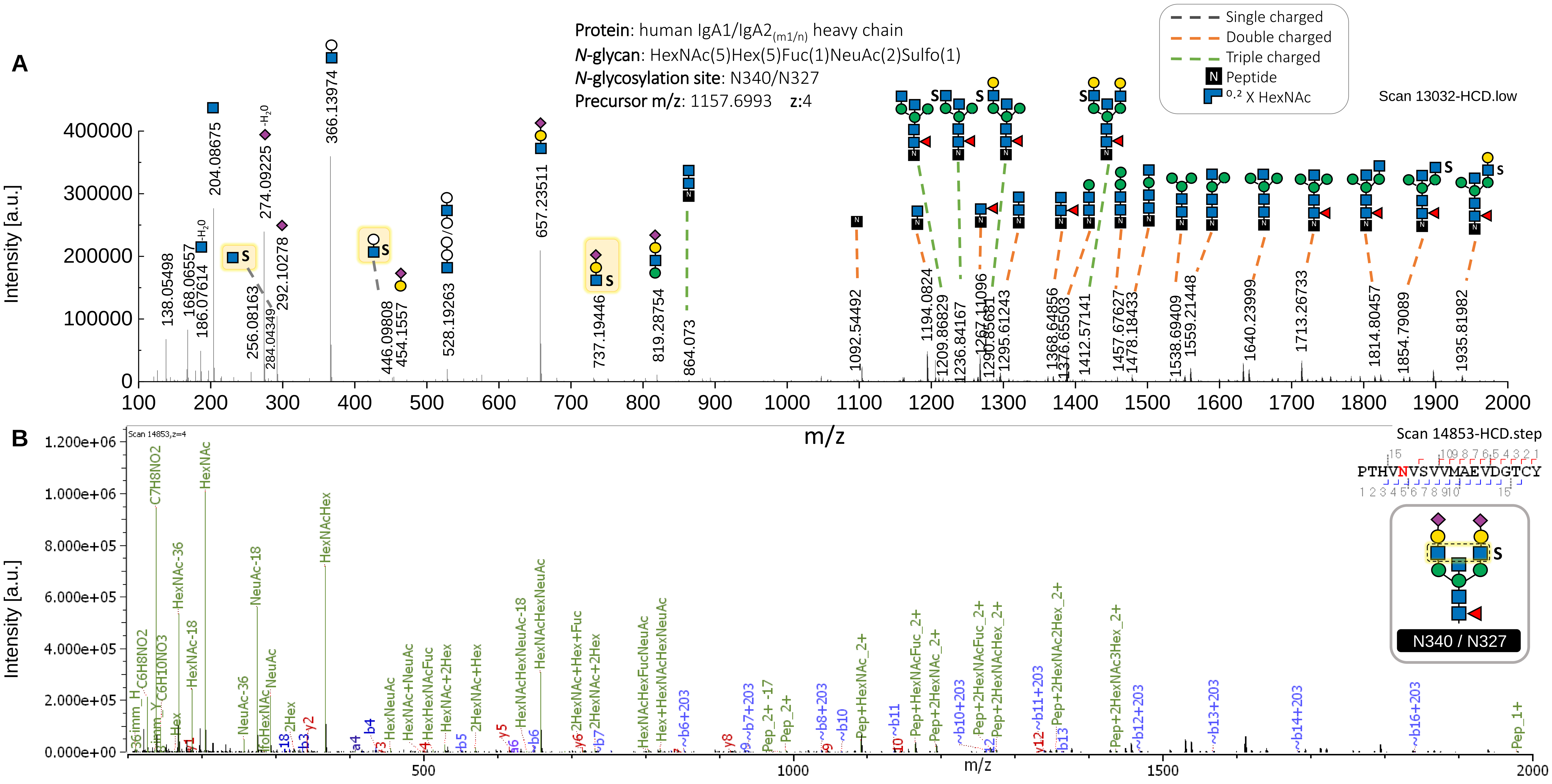

Supplementary Figure 5. Annotated data of a glycopeptide bearing the sulfated *N*-glycan HexNAc(5)Hex(5)Fuc(1)NeuAc(2)Sulfo(1). (A) Description of bisected *N*-glycan structure through HCD.low spectrum shows Y ions indicating sulfation most likely in one antenna HexNAc and not in the bisecting HexNAc. **(B)** The Byonic ™ annotation of the HCD.step spectrum refers to an amino acid sequence of this *N*-glycopeptide containing the homologous site N340-IgA1/N327-IgA2$_{(m1/n)}$. The HCD.step spectrum also shows the oxonium ion confirming HexNAc sulfation ($Sulfo_1HexNAc_1$/284.0439 $[M+H]^+$). The HCD.low and HCD.step spectra that most extensively describe the glycoform and peptide sequence, acquired within the shortest time, were selected for representing this glycopeptide.

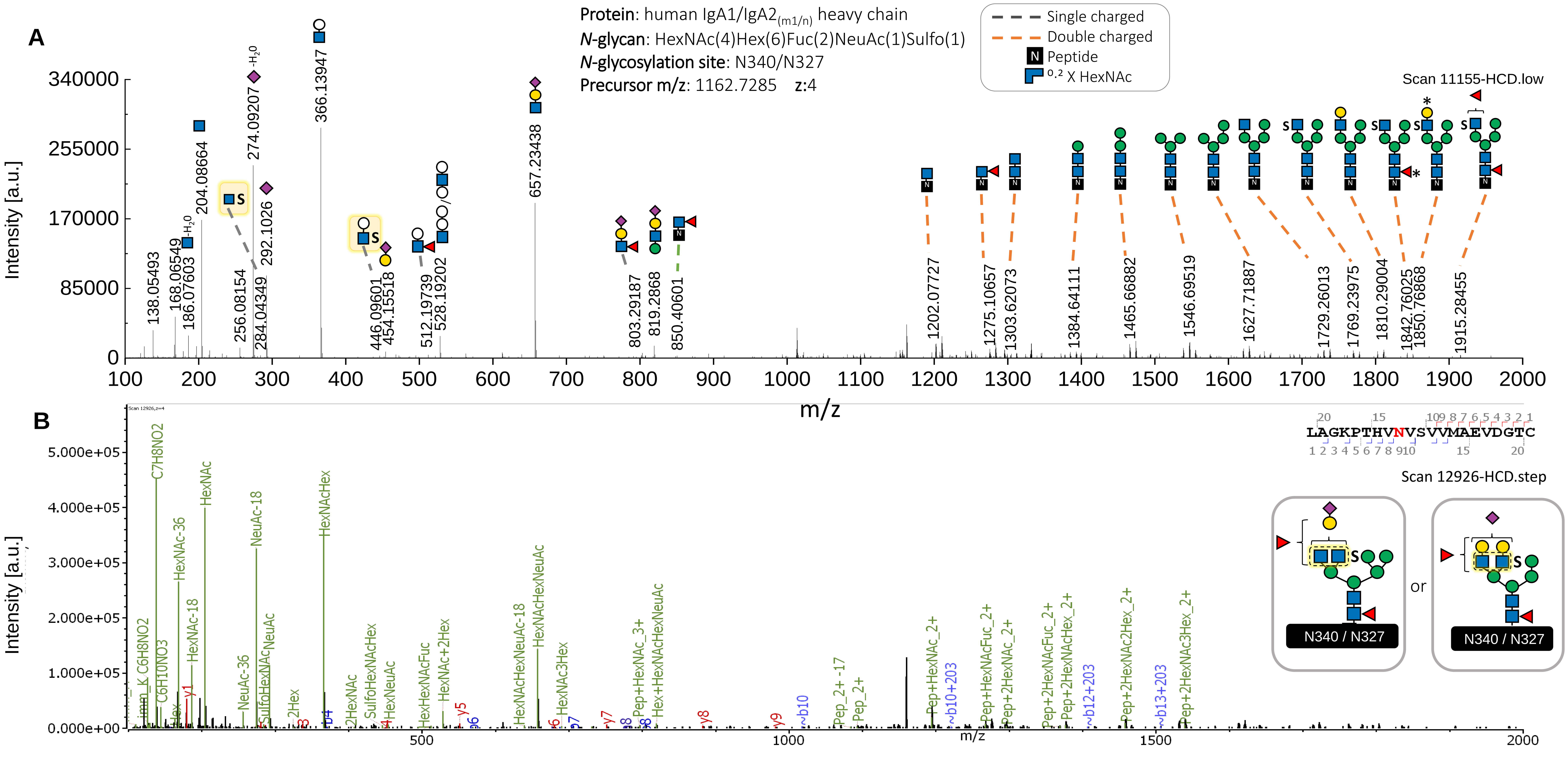

Supplementary Figure 6. Annotated data of a glycopeptide bearing the sulfated *N*-glycan HexNAc(4)Hex(6)Fuc(2)NeuAc(1)Sulfo(1). (A) Description of di-fucosylated hybrid *N*-glycan structure through HCD.low spectrum shows B and Y ions indicating HexNAc sulfation. The *N*-glycan building blocks with an asterisk symbol (*) depict the *N*-glycan composition by a Y ion assigned to the second isotope peak of the expected m/z. **(B)** Byonic ™ annotation of the HCD.step spectrum refers to an amino acid sequence containing homologous site N340-IgA1/N327-IgA2$_{(m1/n)}$ through the b and y fragment ions released from the *N*-glycopeptide. The HCD.step spectrum shows the oxonium marker ions confirming HexNAc sulfation (Sulfo$_1$HexNAc$_1$/284.0433 $[M+H]^+$). The HCD.low and HCD.step spectra that most extensively describe the glycoform and peptide sequence, acquired within the shortest time, were selected for representing this glycopeptide.

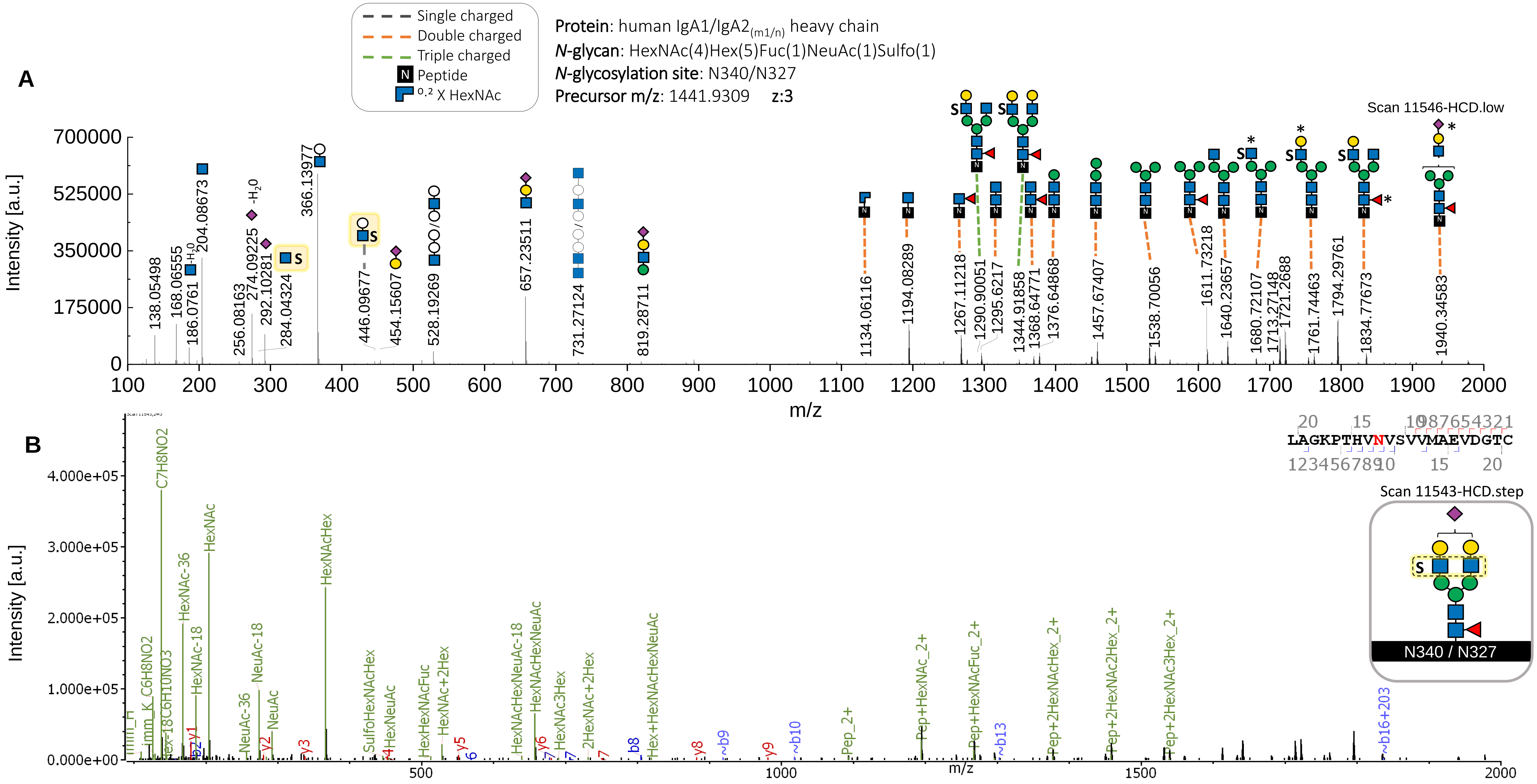

Supplementary Figure 7. Annotated data of a glycopeptide bearing the sulfated *N*-glycan HexNAc(4)Hex(5)Fuc(1)NeuAc(1)Sulfo(1). (A) Description of the *N*-glycan structure through HCD.low spectrum shows B and Y ions confirming one sulfation linked to antenna HexNAc. The *N*-glycan building blocks with an asterisk symbol (*) continue to depict the *N*-glycan composition by a Y ion assigned to the second isotope peak of the expected m/z. **(B)** HCD.step spectrum annotated by Byonic ™ recognizes b and y ions corresponding to an amino acid sequence of this *N*-glycopeptide containing the homologous site N340-IgA1/N327-IgA2$_{(m1/n)}$. The HCD.step spectrum shows the oxonium marker ion $Sulfo_1HexNAc_1Hex_1$/446.0967 $[M+H]^+$ and also contains the oxonium ion $Sulfo_1HexNAc_1$/284.0437 $[M+H]^+$, but the label is not shown due to lack of space. The HCD.low and HCD.step spectra that most extensively describe the glycoform and peptide sequence were selected for representing this glycopeptide.

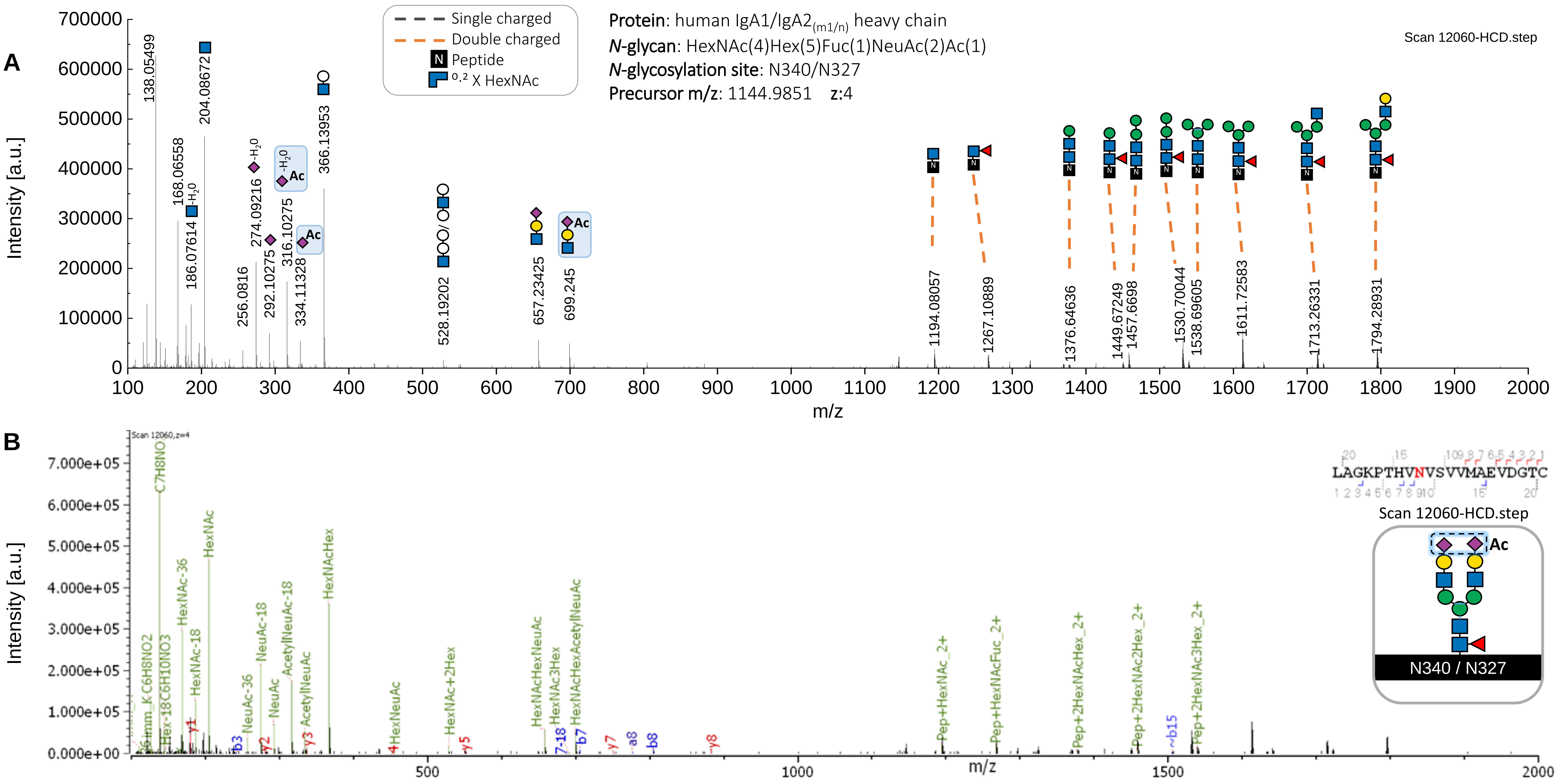

Supplementary Figure 8. Annotated data of glycopeptide containing NeuAc *O*-acetylation in the *N*-glycan HexNAc(4)Hex(5)Fuc(1)NeuAc(2)Ac(1). (A) Description of *N*-glycan structure through HCD.step spectrum shows B and Y ions providing structural evidence on NeuAc *O*-acetylation (highlighted in blue). **(B)** Byonic ™ annotation of the HCD.step spectrum identifies fragment ions released from a peptide containing the homologous site N340-IgA1/N327-IgA2$_{(m1/n)}$. Oxonium marker ions confirming NeuAc *O*-acetylation (Acetyl$_1$NeuAc$_1$/316.1027 [M-H2O+H]$^+$, Acetyl$_1$NeuAc$_1$/334.1133 [M+H]$^+$ and HexNAc$_1$Hex$_1$Acetyl$_1$NeuAc$_1$/699.2454 [M+H]$^+$). The HCD.step spectrum that most extensively describe the glycoform and peptide sequence was selected for representing this glycopeptide.

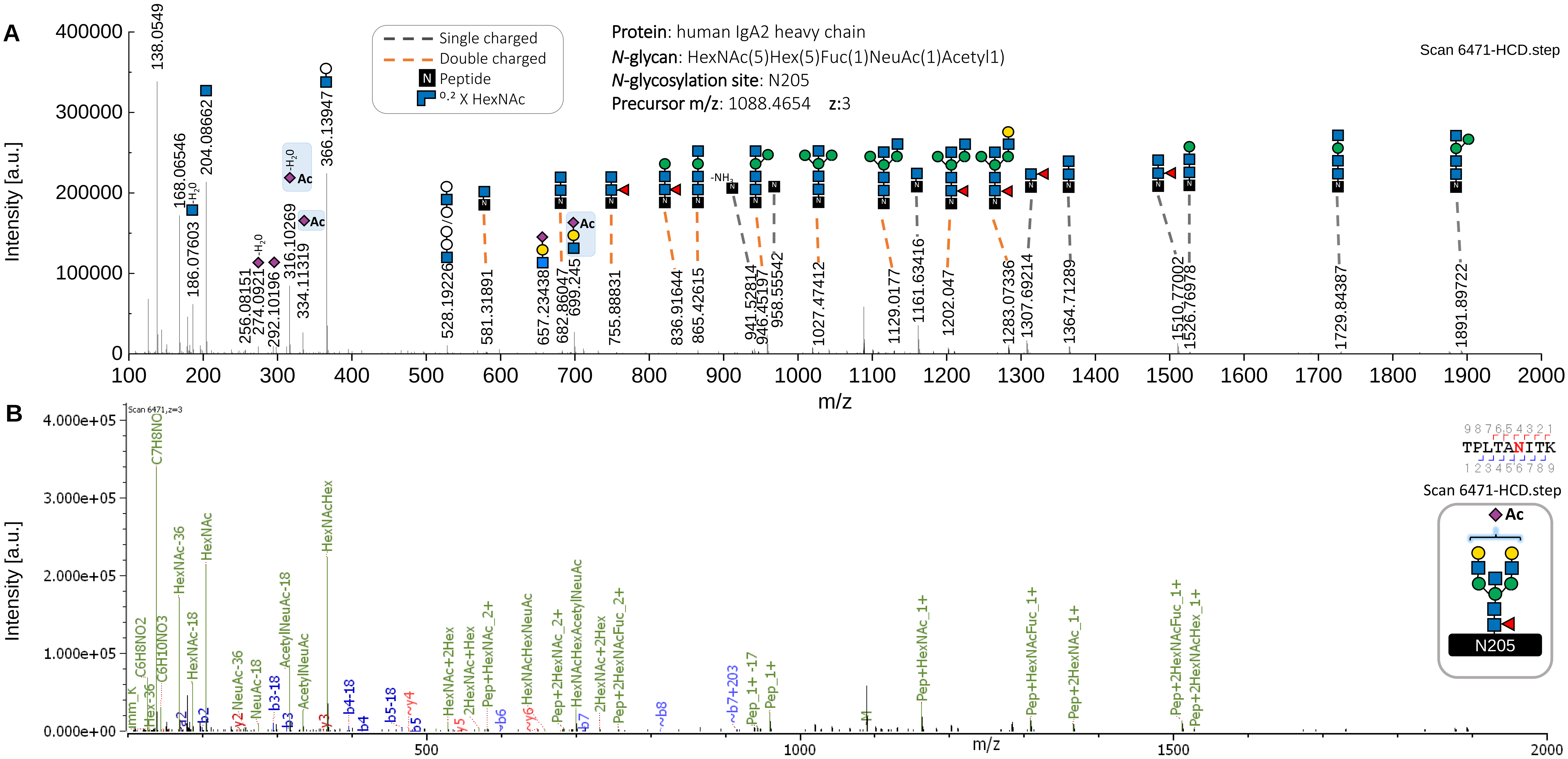

Supplementary Figure 9. Annotated data of glycopeptide containing the NeuAc *O*-acetylated *N*-glycan HexNAc(5)Hex(5)Fuc(1)NeuAc(1)Ac(1). (A) Description of bisected *N*-glycan structure through HCD.step spectrum shows B ions indicating NeuAc *O*-acetylation (highlighted in blue). **(B)** Byonic ™ annotation of the HCD.step spectrum displays b and y ions that identify an *N*-glycopeptide containing the site N205-IgA2$_{(m1/m2/n)}$. Oxonium marker ions confirming NeuAc *O*-acetylation (Acetyl$_1$NeuAc$_1$/316.1027 [M+H-H_2O]$^+$, Acetyl$_1$NeuAc$_1$/334.1132 [M+H]$^+$ and HexNAc$_1$Hex$_1$Acetyl$_1$NeuAc$_1$/699.2450 [M+H]$^+$). The HCD.step spectrum that most extensively describe the glycoform and peptide sequence was selected for representing this glycopeptide.

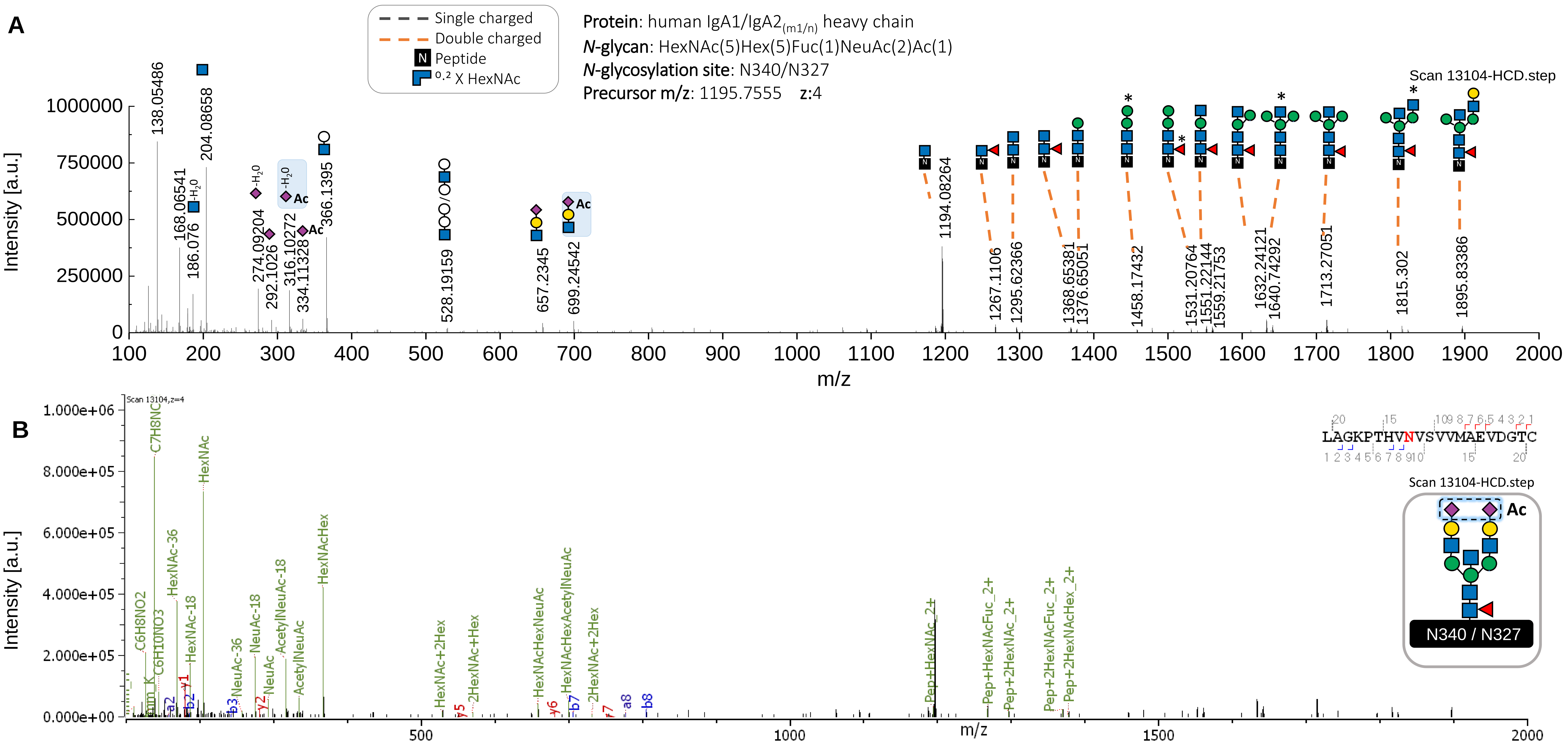

Supplementary Figure 10. Annotated data of glycopeptide containing NeuAc *O*-acetylation in the *N*-glycan HexNAc(5)Hex(5)Fuc(1)NeuAc(2)Ac(1). (A) Description of bisected *N*-glycan structure through HCD.step spectrum shows B ions indicating NeuAc *O*-acetylation (highlighted in blue). The *N*-glycan building block with an asterisk symbol (*) depicts the *N*-glycan composition by a Y ion assigned to the second isotope peak of the expected m/z. **(B)** Byonic ™ annotation of the HCD.step spectrum identifies fragment ions released from an *N*-glycopeptide containing the homologous site N340-IgA1/N327-IgA2$_{(m1/n)}$. Oxonium marker ions confirming NeuAc O-acetylation (Acetyl$_1$NeuAc$_1$/316.1027 [M-H2O+H]$^+$, Acetyl$_1$NeuAc$_1$/334.1133 [M+H]$^+$ and HexNAc$_1$Hex$_1$Acetyl$_1$NeuAc$_1$/699.2454 [M+H]$^+$). The HCD.step spectrum that most extensively describe the glycoform and peptide sequence was selected for representing this glycopeptide.

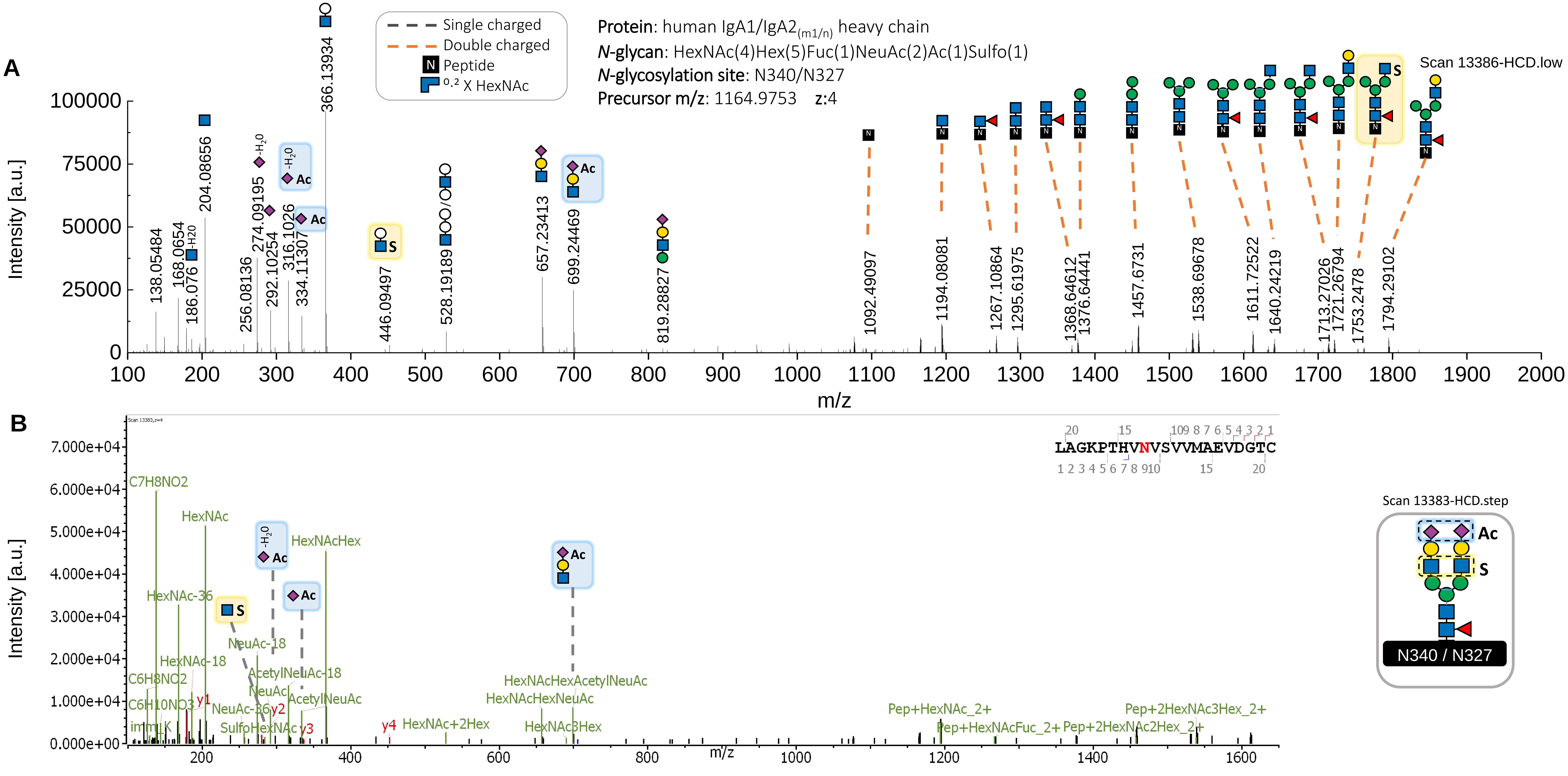

Supplementary Figure 11. Annotated data of a glycopeptide containing NeuAc *O*-acetylation plus HexNAc sulfation in the *N*-glycan HexNAc(4)Hex(5)Fuc(1)NeuAc(2)Ac(1)Sulfo(1). **(A)** Description of *N*-glycan structure through HCD.low spectrum shows B and Y ions providing structural evidence on NeuAc *O*-acetylation (highlighted in blue) and antenna sulfation (highlighted in yellow). **(B)** Byonic ™ annotation of the HCD.step spectrum identifies fragment ions released from an *N*-glycopeptide containing the homologous site N340-IgA1/N327-IgA2$_{(m1/n)}$. In the HCD.step spectrum, the oxonium ion $Sulfo_1HexNAc_1$/284.0431 $[M+H]^+$ confirms HexNAc sulfation. NeuAc *O*-acetylation is confirmed by the oxonium ions: $Acetyl_1NeuAc_1$/316.1027 $[M-H2O+H]^+$, $Acetyl_1NeuAc_1$/334.1133 $[M+H]^+$ and $HexNAc_1Hex_1Acetyl_1NeuAc_1$/699.2454 $[M+H]^+$. The configuration of the exported Byonic image was specially set to display these key oxonium ions.

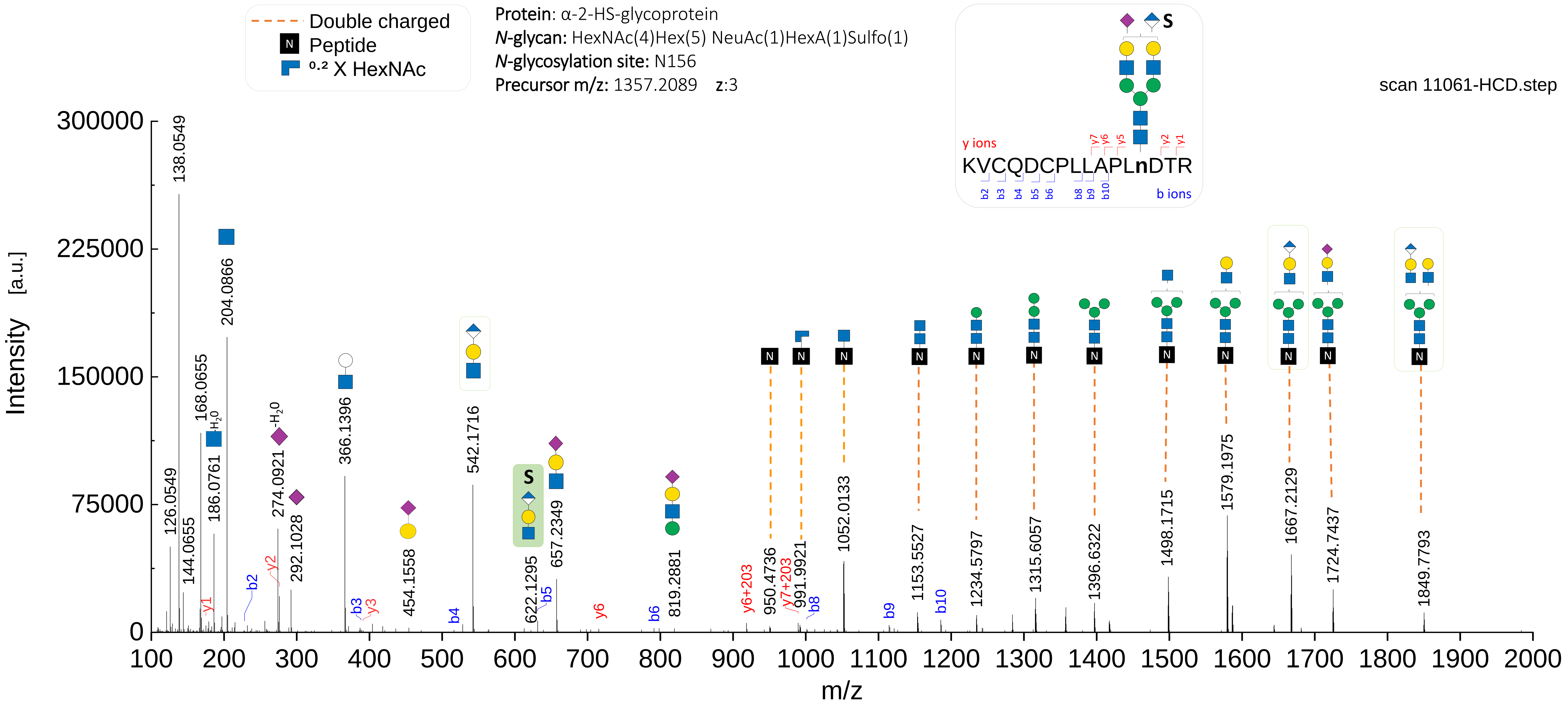

Supplementary Figure 12. Annotated data of a glycopeptide potentially containing HexA sulfation in the *N*-glycan HexNAc(4)Hex(5)NeuAc(1)HexA(1)Sulfo(1) of a contaminant protein. Annotation of *N*-glycan structure through HCD.step spectrum shows B and Y ions providing structural evidence on HexA as one antenna capping sugar (fragment ions framed in green color). The oxonium marker ion confirming HexA sulfation ($HexNAc_1Hex_1HexA_1Sulfo_1$/622.1295 $[M+H]^+$, highlighted in green) resembles the glycoepitope human natural killer-1 (HNK-1) composed of glucuronic acid. The b and y fragment ions (in blue and red color, respectively) demonstrate a tryptic peptide bearing the site N156 in α-2-HS-glycoprotein. The *N*-glycopeptide was identified through Byonic ™. The HCD.step spectrum that most extensively describe the glycoform and peptide sequence was selected for representing this glycopeptide.

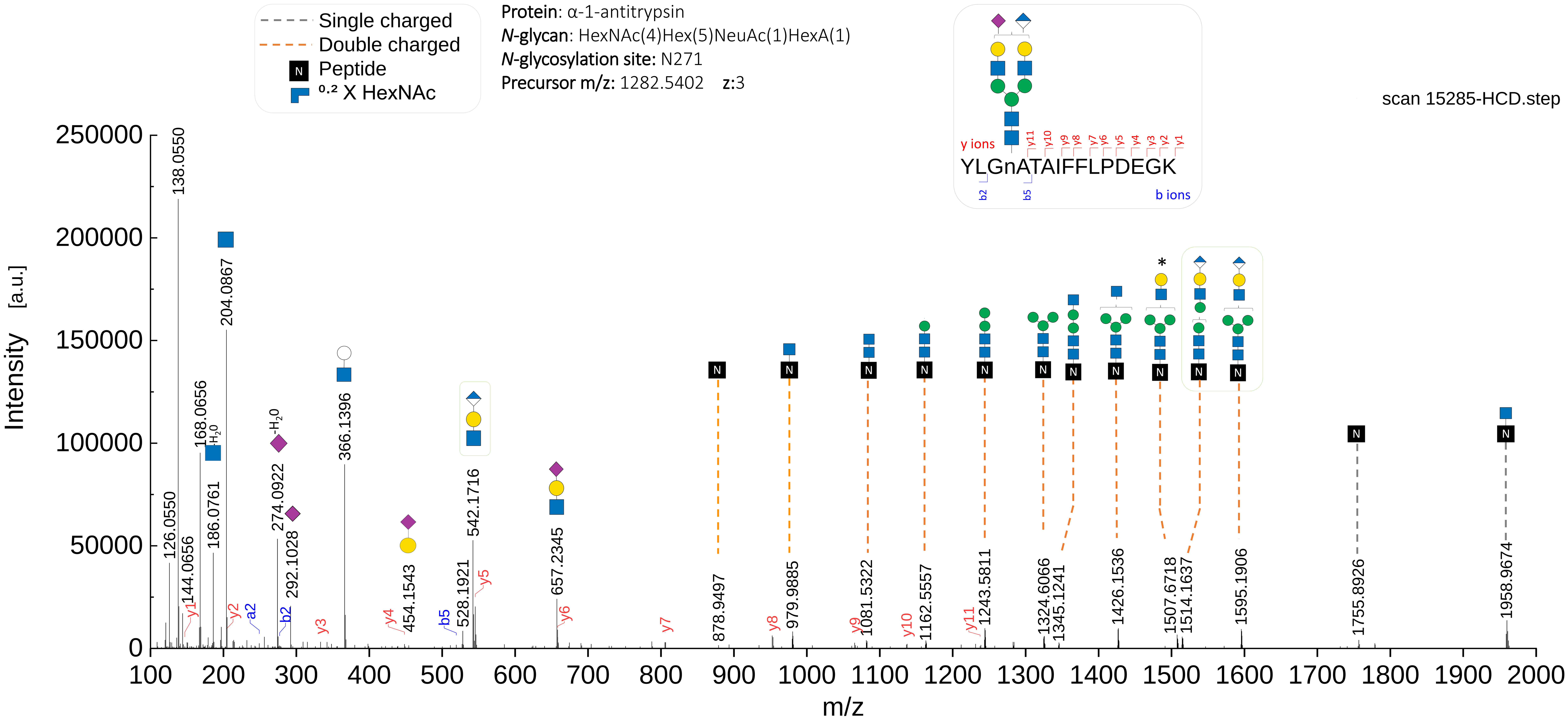

Supplementary Figure 13. Annotated data of a glycopeptide potentially containing HexA in the *N*-glycan HexNAc(4)Hex(5)NeuAc(1)HexA(1) of a contaminant protein. Annotation of *N*-glycan structure through HCD.step spectrum shows B and Y ions providing structural evidence on HexA as one antenna capping sugar (fragment ions framed in green color). The oxonium marker ion confirming HexA ($HexNAc_1Hex_1HexA_1$/542.1716 $[M+H]^+$) resembles the non-sulfated form of the human natural killer-1 glycoepitope (HNK-1). The b and y fragment ions (in blue and red color, respectively) demonstrate a tryptic peptide bearing the site N271 in α-1-antitrypsin. The *N*-glycopeptide was identified through Byonic ™. The HCD.step spectrum that most extensively describe the glycoform and peptide sequence was selected for representing this glycopeptide. The *N*-glycan building blocks with an asterisk symbol (*) continue to depict the *N*-glycan composition by a Y ion assigned to the second isotope peak of the expected m/z.
